# Neuromuscular Development and Disease: Learning From *in vitro* and *in vivo* Models

**DOI:** 10.3389/fcell.2021.764732

**Published:** 2021-10-27

**Authors:** Zachary Fralish, Ethan M. Lotz, Taylor Chavez, Alastair Khodabukus, Nenad Bursac

**Affiliations:** Department of Biomedical Engineering, Pratt School of Engineering, Duke University, Durham, NC, United States

**Keywords:** tissue engineering, induced pluripotent stem cells, disease modeling, neuromuscular junction, human skeletal muscle, muscular dystrophy, drug development, organ on a chip

## Abstract

The neuromuscular junction (NMJ) is a specialized cholinergic synaptic interface between a motor neuron and a skeletal muscle fiber that translates presynaptic electrical impulses into motor function. NMJ formation and maintenance require tightly regulated signaling and cellular communication among motor neurons, myogenic cells, and Schwann cells. Neuromuscular diseases (NMDs) can result in loss of NMJ function and motor input leading to paralysis or even death. Although small animal models have been instrumental in advancing our understanding of the NMJ structure and function, the complexities of studying this multi-tissue system *in vivo* and poor clinical outcomes of candidate therapies developed in small animal models has driven the need for *in vitro* models of functional human NMJ to complement animal studies. In this review, we discuss prevailing models of NMDs and highlight the current progress and ongoing challenges in developing human iPSC-derived (hiPSC) 3D cell culture models of functional NMJs. We first review *in vivo* development of motor neurons, skeletal muscle, Schwann cells, and the NMJ alongside current methods for directing the differentiation of relevant cell types from hiPSCs. We further compare the efficacy of modeling NMDs in animals and human cell culture systems in the context of five NMDs: amyotrophic lateral sclerosis, myasthenia gravis, Duchenne muscular dystrophy, myotonic dystrophy, and Pompe disease. Finally, we discuss further work necessary for hiPSC-derived NMJ models to function as effective personalized NMD platforms.

## Introduction

Neuromuscular diseases (NMDs) are a broadly defined group of disorders that lead to progressive impairment of motor function. The NMDs primarily involve dysfunction of motor neurons (MNs), skeletal muscle (SkM), or their synaptic connection, the neuromuscular junction (NMJ). Different NMDs have distinct tissue origins such as MNs [e.g., amyotrophic lateral sclerosis (ALS) either from direct loss or retrograde degeneration of MNs], muscle [e.g., Duchenne muscular dystrophy (DMD) or myotonic dystrophy (DM)], NMJs [e.g., myasthenia gravis (MG) or congenital myasthenic syndromes (CMS)], or a combination thereof (e.g., Pompe disease). Regardless of the origin, the structural and/or functional deficit in a targeted tissue will resonate throughout the entire motor unit, leading to multiple shared symptoms among different NMDs. NMDs are estimated to affect 160 per 100,000 people worldwide (Deenen et al., 2015); however, despite this high prevalence, outcomes are often fatal as few curative treatments are available. Therefore, comprehensive biomimetic and clinically predictive *in vitro* and *in vivo* NMD models are essential for accelerating our understanding of the underlying disease mechanisms and development of effective therapeutics (Babin et al., 2014; Aartsma-Rus and van Putten, 2019). Animal models have been invaluable to our current understanding of NMDs as they capture important clinical features of the disease. Still, currently available animal models do not fully recapitulate the diverse range of disease phenotypes nor disease severity due to the complex genetic and non-genetic nature of human NMDs.

In recent years, high clinical and genetic heterogeneity of NMDs has prompted the considerations of personalized approaches to study and treat these devastating diseases. Historically, investigations of human NMDs have been hindered due to difficulty imaging NMJs *in vivo*, limited capability to isolate neural stem cells, and the postmitotic nature of adult MNs complicating *in vitro* studies. Over the past decade, advances in human induced pluripotent stem cell (hiPSC) technology, have provided a novel source of human somatic cells for pre-clinical research. hiPSCs can be generated from ethical and accessible sources, such as the skin and blood, and function as a potentially unlimited, patient-specific source of traditionally inaccessible cells such as MNs and cardiomyocytes. Additionally, hiPSC-derived cells can be integrated into two- (2D) and three-dimensional (3D) culture systems to enable novel studies of human development, disease, and pharmacology. hiPSC-based derivations of MNs and SkM, in particular, provide easily accessible, highly expandable sources of patient-specific NMD-relevant tissues. Notably, these platforms complement animal models creating an efficient and predictive system for patient specific NMD modeling and drug development. Incorporation of hiPSC-derived MNs and SkM into 3D cultures and organ-on-a-chip systems adds necessary structural complexity and genetic and environmental control over the cell-specific behavior. These tissue-engineered motor units recapitulate the nature of functional NMJs offering potential for improved mechanistic understanding of complex NMDs (Osaki et al., 2018; Bakooshli et al., 2019; Vila et al., 2019; Faustino Martins et al., 2020; Rimington et al., 2021).

In this review, we first explore the individual components of the NMJ including how they interact and contribute to NMJ functionality. We then compare current animal and *in vitro* hiPSC models of NMDs, focusing on ALS, MG, DMD, DM, and Pompe disease. We end by discussing the future of NMD modeling and strategies to address limitations in creating *in vitro* functional motor units that would allow predictive, patient-specific studies and treatment of NMDs.

## Development

Understanding embryonic development of NMJs and their physiological roles is important to critically analyze methods to derive relevant cell types from hiPSCs and to compare biomimetic nature and effectiveness of the current and future tissue-engineered NMD models. Therefore, we provide an overview of MN, SkM, and Schwann cell (SC) development and draw parallels to current methods to differentiate these cells from hiPSCs. We finalize this section by describing NMJ development and function.

### Motor Neuron Development

Over the past decade, methods to differentiate hiPSCs into neuronal cells has rapidly progressed due to increased understanding of early neural development and commitment of neuroprogenitor cells to highly specialized neural subtypes, including MNs (Deenen et al., 2015). MNs are found throughout the CNS and can be divided into upper MNs (UMNs) or lower MNs (LMNs), which, despite their shared nomenclature, are developmentally and genetically distinct. Different NMDs, such as primary lateral sclerosis, progressive muscular atrophy, or ALS, can target either or both of UMNs and LMNs (Liewluck and Saperstein, 2015) making their distinction an important consideration when modeling NMDs. UMNs originate from the pre-motor and primary motor regions of the cerebral cortex. Their axons form glutamatergic connections with LMNs located in the brainstem and ventral horn of the spinal cord. Axons of LMNs project beyond the CNS forming cholinergic synapses with multiple tissue types to control a wide variety of physiological processes. As a result of these regional differences, the genetic and molecular events leading to MN development diverge early. Therefore, it is important to consider the development of the CNS in its entirety and understand the molecular mechanisms underlying MN diversity to develop physiologically relevant models for NMDs.

**FIGURE 1:**
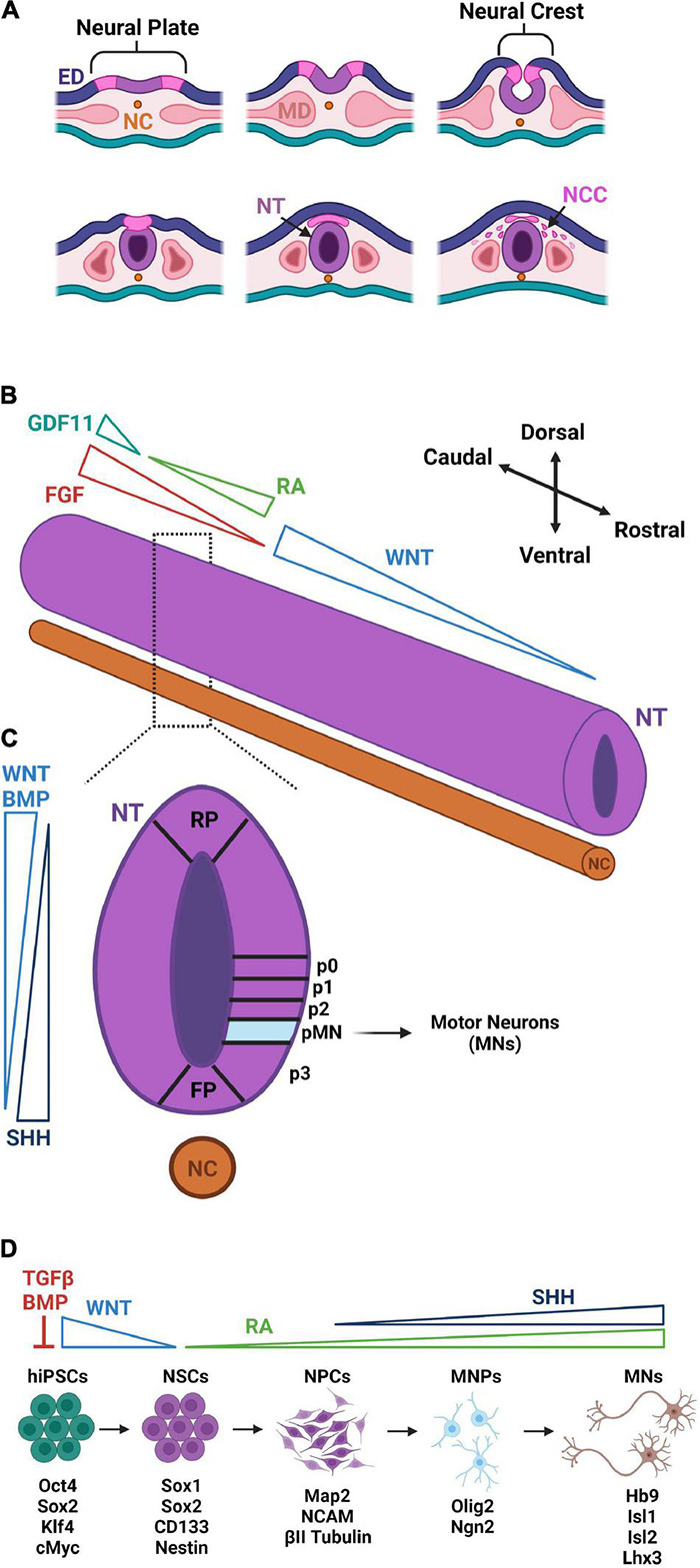
Early development and hiPSC-based differentiation of motor neurons. **(A)** After the notochord (NC) signals inward folding of the ectoderm at the neural plate, the neural crest is brought together forming the neural tube (NT). The neural crest then forms neural crest cells (NCCs) which differentiate in the peripheral nervous system. The cells of mesoderm (MD) differentiate into somites and, eventually, the musculoskeletal system. **(B)** Along the rostral–caudal axis of the NT, WNT gradients dictate regionalization of the brain and RA/FGF/GDF11 gradients dictate segmentation of the spinal cord. **(C)** Along the dorsal–ventral axis of the NT, antithetical WNT/BMP [derived from the roof plate (RP)] and SHH gradients [derived from the floor plate (FP)] dictate patterning and the formation of the five ventral progenitor cell domains (p0, p1, p2, pMN, and p3). The pMN domain is the source of subsequent MN specification. **(D)** hiPSC-derivations of MNs begin with dual SMAD inhibition of TGFβ and BMP pathways to trigger neural stem cell (NSC) differentiation. WNT and RA signaling direct NSC differentiation into neural progenitor cells (NPCs) of the spinal cord region. With the addition of SHH signaling, NPCs further differentiate to Olig2-expressing MN progenitors (MNPs). Suppression of Olig2 and upregulation of Ngn2 commit MNPs to a post-mitotic MN lineage that express Hb9, Isl1, Isl2, and Lhx3. Distinct colors are used to denote approximate correspondence between stages of hiPSC differentiation in panel **(D)** and embryonic development in panel **(C)**.

Cells of the developing vertebrate nervous system are derived from the ectoderm which forms during gastrulation (Figures 1A–C). Inhibition of TGFβ and FGF initiates neurulation causing the ectoderm to fold inward generating three new regions: (1) neural tube, (2) neural crest, and (3) external ectoderm (Ozair et al., 2013). Each region contains cell progenies restricted to a limited number of distinct fates, and those found in the neural tube are destined to form the brain and spinal cord. Signaling molecules from the mesodermal notochord coordinate the formation of the neural tube in the proper spatial orientation along the rostral–caudal and dorsal–ventral axes (Muhr et al., 1999). After neurulation, morphogen production and its subsequent spatio-temporal organization along the two axes causes axial patterning of the neural tube responsible for regional specification of neural subtypes (Wichterle et al., 2002; Li et al., 2005).

Regionalization is first specified in the brain with cells assuming a rostral forebrain identity in the absence of morphogens. These cells continue to develop into the neurons comprising the telencephalic region of the brain, which houses the cerebral cortex where mature UMNs reside (Watanabe et al., 2005). The remaining cells are driven caudally in response to a WNT gradient established by dorsal roof plate cells. This gradient dictates caudal forebrain, midbrain, and hindbrain identities (Nordstrom et al., 2002). LMNs with a distinct spinal character require further caudalization. A major contributing factor to this LMN caudalization is retinoic acid (RA) signaling that leads to rostral identities of the cervical and upper thoracic spinal segments. Presomitic cells of the surrounding paraxial mesoderm convert retinaldehyde to RA via their expression of aldehyde dehydrogenase 1 A2 (ALDH1A2) (Liu et al., 2001). Decreased ALDH1A2 expression is found caudally and corresponds to decreased RA signaling (Liu et al., 2001) and increased FGF signaling, which govern the caudalization of neural precursors to identities of thoracic and lumbar spinal segments (Irioka et al., 2005). High expression of FGF alongside an increasing gradient of GDF11 dictate pattern a sacral spinal identity associated with the most caudal region of the developing spinal cord (Diez del Corral and Storey, 2004). Differential expression of HOX-family genes corresponds to cervical (HOX4 – HOX6), thoracic (HOX8 and HOX9), and lumbar (HOX10 and HOX11) positional identities along the spinal cord (Dasen and Jessell, 2009).

Neural precursors are also subject to dorsal–ventral patterning concurrent to rostral–caudal patterning. WNTs and BMPs derived from roof plate cells mediate dorsal patterning (Son et al., 2011). In contrast, exposure to increasing concentrations of sonic hedgehog (SHH) secreted by floor plate cells drives ventral patterning (Ericson et al., 1996). Dorsal–ventral patterning for the development of UMNs remains poorly understood. On the other hand, ventral positioning of LMN progenitors is known to require a coordinated balance between the antagonizing effects of BMPs/WNTs and SHH (Jessell, 2000). The ventral spinal cord consists of five domains that further restrict neural progenitors to a specific lineage. Interestingly, the MN progenitor (pMN) domain required for LMN specification is also required for oligodendrocyte specification (Ravanelli and Appel, 2015). OLIG2 expression is the earliest marker used to identify progenitors committed to the pMN domain (Ravanelli and Appel, 2015). Over time, oligodendrocyte progenitors will continue to express OLIG2 while committed MN progenitors will begin to express NGN2 which represses OLIG2 expression (Ravanelli and Appel, 2015). Continued expression of NGN2 induces HB9, signifying the formation of a post-mitotic MN (Lee et al., 2009).

Lower motor neurons at this stage are referred to as having a general character until organized into distinct motor columns that correspond to targeted regions of innervation. These regions include the median motor column (MMC), which innervates axial SkM, spinal accessory column (SAC), which innervates the branchial SkM of the face and neck, phrenic motor column (PMC), which innervates the SkM of the diaphragm, lateral motor column (LMC), which innervates appendage muscle, hypaxial motor column (HMC), which innervates intercostal and abdominal SkM, and preganglionic motor column (PGC), which synapse onto ganglionic neurons of the autonomic nervous system (ANS) (Nicolopoulos-Stournaras and Iles, 1983). Each motor column is organized along the rostro-caudal axis and is identified by a unique gene signature (Francius and Clotman, 2014).

### Generation of Motor Neurons From Pluripotent Stem Cells

Over the past decade, a variety of protocols have been used to derive MNs from hiPSCs. These methods vary in length and efficiency; however, they are designed on the genetic and molecular principles of embryonic development (Figure 1D). Historically, neural induction was performed in suspended aggregates of hiPSCs, called embryoid bodies (EBs), in serum-free media without exogenous morphogens. Under these conditions, differentiating hiPSCs undergo spontaneous FGF and BMP inhibition, which naturally guides their differentiation to a neural fate (LaVaute et al., 2009). These early protocols were long and inefficient, often producing high experimental variability. Dramatic improvements came with the discovery that early inhibition of BMP and TGFβ signaling through SMAD inhibition selectively blocks the formation of mesodermal and endodermal cell fates leading to higher percentages of PAX6 and SOX2 expressing neural progenitors at earlier time points (Chambers et al., 2009). Based upon this discovery, dual-SMAD inhibition is now standard practice for the neuralization of hiPSCs in both monolayer cultures as well as EBs. The small molecule, SB431542, is the most utilized TGFβ inhibitor, and is commonly paired with a small molecule inhibitor of BMP (LDN193189, DMH1, or dorsomorphin) or recombinant Noggin, a naturally occurring BMP inhibitor.

After neuralization, neural precursors are committed to MN progenitors by following common patterning principles of caudalization and ventralization. MN differentiation efficiency and culture length have been improved by optimizing the concentrations and timing of patterning morphogens like WNTs, BMPs, RA, FGFs, and SHH (Nordstrom et al., 2002). Although experimental reproducibility has been improved, the variability and length of derivation protocols has remained a challenge. Specifically, the general application of RA and SHH for MN differentiation has been inefficient with yields ranging between 30 and 60% over a culture duration of 21–40 days (Hu and Zhang, 2009; Hester et al., 2011). Early activation of WNTs with CHIR99021 (CHIR) was shown to significantly improve MN differentiation efficiency and speed resulting in an 80% yield in 14 days (Maury et al., 2015). Moreover, when CHIR was continuously added throughout the differentiation, 90% of cells became mature MNs within 12 days (Du et al., 2015). This continued activation of WNTs stabilized excessive ventralization, maintaining a higher population of cells in the pMN domain and reducing the population of NKX2.2 expressing interneuron progenitors of the p3 domain (Du et al., 2015). While expedited protocols for MN generation may benefit cell manufacturing and screening, how accurately “fast-tracked” methods recapitulate adult MN cell physiology and maturation remains unclear.

### Skeletal Muscle Development

**FIGURE 2:**
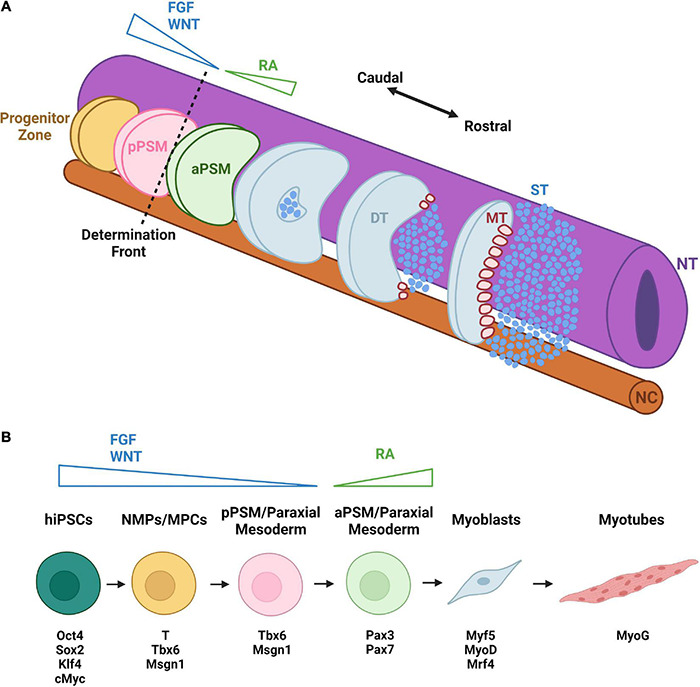
Early development and hiPSC-based differentiation of skeletal muscle. **(A)** Caudal-rostral development of SkM occurs bilaterally along the neural tube (NT) and notochord (NC). From the progenitor zone, cells migrate to the posterior presomitic mesoderm (pPSM) with a decreasing gradient of FGF and WNT signals. They then cross the determination front to enter the anterior presomitic mesoderm (aPSM). With increasing retinoic acid (RA) gradient, somite formation begins. As cells continue to travel rostrally, the dermomyotome (DT), sclerotome (ST), and myotome (MT) form, initiating primary myogenesis. **(B)** hiPSC differentiation to skeletal muscle begins with WNT and FGF activation, inducing a shift into neuromesodermal progenitors (NMPs) and then muscle progenitor cells (MPCs) expressing the transcription factors T, Tbx6, and Msgn1. Subsequent loss of T expression leads to formation of paraxial mesoderm cells resembling skeletal muscle progenitors of pPSM. Through RA activation, these muscle progenitors begin to express the muscle stem cell markers Pax3 and Pax7 and eventually differentiate into myoblasts expressing early myogenic markers Myf5, MyoD, and Mrf4. The myoblasts can fuse into myotubes that express the late muscle differentiation marker MyoG. Distinct colors are used to denote approximate correspondence between stages of hiPSC differentiation in panel **(B)** and embryonic development in panel **(A)**.

Development of skeletal muscle (SkM) begins with the paraxial mesoderm (Wachtler, 1992). The paraxial mesoderm (PM) forms in the primitive streak/blastopore during gastrulation and is comprised of two bilateral strips of presomitic mesoderm (PSM) flanking the neural tube and notochord (White et al., 2005; Figure 2A). Cells acquiring the PM fate require suppression of BMP signaling *in vivo* (Winnier et al., 1995). In the posterior compartments of the PM, there is an unsegmented progenitor zone comprised of neuromesodermal progenitors (NMP) (Tzouanacou et al., 2009) and other progenitor cells which give rise to the paraxial mesoderm, neural tube derivatives, lateral plate derivatives, and notochord (Takemoto et al., 2011; Garriock et al., 2015). Cells within the progenitor zone develop into skeletal muscle progenitor cells as a result of WNT and FGF signaling gradients which target transcription factors essential for PSM specification and patterning such as brachyury (T), Tbx6, and Msgn1 (Ciruna and Rossant, 2001; Nowotschin et al., 2012). Differentiated cells acquire the identity of mesoderm progenitor cells (MPCs) within the most posterior region of the PSM (Chalamalasetty et al., 2014).

In the next stage of development, MPCs attain the posterior PSM (pPSM) fate characterized by the downregulation of *T* and expression of *Msgn1* and *Tbx6* (Chalamalasetty et al., 2014). In the posterior two-thirds of the PSM, MPCs and pPSMs experience oscillations of the segmentation clock (pulses of Notch, FGF, and WNT signaling) to control the production of somites (Dubrulle et al., 2001; Aulehla et al., 2003). As skeletal muscle cells continue to develop, reach the determination front then enter the anterior third of the PSM (Dubrulle et al., 2001). At the determination front, the oscillations of the segmentation clock cease, *Msgn1* is downregulated, and *Pax3, Mesp2, Foxc1/2*, and *Meox1/2* genes are upregulated (Kume et al., 2001; Mankoo et al., 2003). Within the anterior PSM, retinoic acid (RA) counteracts the WNT and FGF signaling (Sakai et al., 2001). Furthermore, a posterior fissure forms at the junction between *Mesp2*^+^ and *Mesp2*^–^ cells to create new somites (Dubrulle et al., 2001; Aulehla et al., 2003). Shortly after somites are formed, they become subdivided into ventral mesenchymal sclerotome and dorsal epithelial dermomyotome, the latter of which contains SkM (alongside dermis and brown fat) progenitors and maintains *Pax3* expression (Lepper and Fan, 2010; Sanchez-Gurmaches and Guertin, 2014). Soon after its formation, primary myogenesis begins when dorsally located dermomyotomal cells lose Pax3 expression and upregulate myogenic factor *Myf5* (Ott et al., 1991). These early myogenic cells delaminate from the dermomyotome and contribute to the formation of the first embryonic muscles—myotomes (Denetclaw et al., 1997). Myogenesis then officially commences with primary myogenesis and the generation of primary myofibers that serve as the foundation for adult muscle formation.

During secondary myogenesis, myogenic progenitors expressing transcription factor *Pax7* sustain muscle growth by fusing among themselves or to existing primary myofibers generating β-enolase expressing secondary or fetal myofibers (Fougerousse et al., 2001). A subset of these Pax7+ progenitors localize under the basal lamina where the eventually become satellite cells which contribute to the repair and regeneration of damaged muscle fibers in adults (Dumont et al., 2015a). During muscle fiber maturation, expression of embryonic myosin heavy chain changes to adult myosin heavy chain isoforms with oxidative, slow twitch or glycolytic, fast twitch fiber phenotypes (Khodabukus, 2021). Additionally, actin and myosin assemble into sarcomeres, sarcomeres assemble into myofibrils, NMJs are formed at the sarcolemma, and triads are established from a network of tubules for facilitated neural excitation (Pourquié et al., 2018). Secondary and later stages of myogenesis are controlled by TGFβ (Gu et al., 2016), hepatocyte growth factor (HGF) (Bladt et al., 1995), WNT (van der Velden et al., 2006), and insulin-like growth factor (IGF) (Chargé and Rudnicki, 2004) signaling. The final phases of myogenesis are controlled by transcription factors including *Myf5*, *MyoD*, *Myomaker*, and *MyoG* to facilitate the fusion of muscle progenitor cells, hypertrophy of myofibers, and innervation by MNs to generate fully functional SkM (Zhang et al., 2020).

### Generation of Skeletal Muscle From Pluripotent Stem Cells

The derivations of SkM from hiPSCs typically fall under one of two major approaches (Kodaka et al., 2017; Jiwlawat et al., 2018). The first approach involves transgene-based approaches whereby hiPSCs are directly reprogrammed into myogenic progenitor cells through overexpression of muscle specific transcription factors (Kodaka et al., 2017). The second approach involves transgene-free methods whereby developmental myogenesis in hiPSCs is recapitulated through the administration of small molecules, such as FGF2 and GSK3β inhibitor, which activate or inhibit myogenic signaling pathways (Jiwlawat et al., 2018; Figure 2B).

The transgene-based approaches generate myogenic progenitors from hiPSCs or their mesodermal derivatives by transient or constitutive overexpression of master regulators of myogenesis, such as PAX7 (Darabi et al., 2012; Rao et al., 2018) or MYOD1 (Abujarour et al., 2014; Albini and Puri, 2014; Maffioletti et al., 2015). Overexpression of exogenous myogenic genes has been accomplished through mRNA transfection (Warren et al., 2010), as well as transduction with adenoviral (Goudenege et al., 2012) or lentiviral (Albini and Puri, 2014; Maffioletti et al., 2015; Rao et al., 2018) vectors. Through these transdifferentiation methods, as many as 90% of cells commit to a myogenic identity and can differentiate into SkM progenitor cells (Tanaka et al., 2013; Abujarour et al., 2014; Rao et al., 2018). Use of fluorescence reporter genes co-expressed with transcription factors can allow further cell purification by fluorescence-activated cell sorting (FACS) (Darabi et al., 2012; Rao et al., 2018). Alternatively, activation of endogenous transcription factors (e.g., Pax7) using CRISPR/Cas9 methodology can lead to stable epigenetic reprogramming of hiPSCs and generation of myogenic progenitor cells (Kwon et al., 2020). The resulting SkM progenitors derived using transgene-based approaches survive and function when implanted in immunocompromised mice (Darabi et al., 2012; Kwon et al., 2020), while 3D engineered tissues generated from these cells can become functional muscle with the ability to survive and function *in vivo* (Rao et al., 2018). Nevertheless, these differentiation methods do not reflect normal development and despite the ability to obtain large numbers of human myogenic progenitors, regulatory concerns regarding genetic modification of cells may limit their potential therapeutic use (Jiwlawat et al., 2018).

A second approach, known as directed differentiation, mimics myogenic development through sequential addition of small molecules to activate or suppress specific signaling pathways. For example, CHIR-99021 activates WNT signaling through GSK3β inhibition, LDN-193189 inhibits BMP signaling, and HGF and IGF1 activate their respective signaling pathways (Chal et al., 2016). Despite no genetic modification and reliance on natural developmental ques, directed differentiation protocols require significantly longer culture time and exhibit considerably lower yields and higher heterogeneity of myogenic cells compared to transgene-based methods (Kodaka et al., 2017; Jiwlawat et al., 2018). Purity of myogenic progenitors can be increased by sorting for cell surface markers such as CDH13 (Nalbandian et al., 2021), FGFR4 (Nalbandian et al., 2021), ERBB3 (Hicks et al., 2018), and NGFR (Hicks et al., 2018), however, use of FACS further decreases cell yield. Recent protocols for expansion and cryopreservation of FACS-sorted hiPSC-derived myogenic progenitors may offer means to obtain clinically relevant cell quantities (van der Wal et al., 2018).

Recent transcriptomic analyses have shown that hiPSC-derived myogenic progenitors are developmentally immature and arrested between embryonic and fetal muscle stem cell stages (Xi et al., 2020; Nayak et al., 2021). Nevertheless, they can successfully fuse into myotubes that exhibit key functional behaviors of SkM, including generation of calcium transients and contractile force and robust response to acetylcholine (Skoglund et al., 2014; Rao et al., 2018), albeit at lower levels compared to primary human myotubes (Rao et al., 2018). Further advances in maturity of hiPSC-derived SkM cells will lead to improved modeling of human NMDs *in vitro*.

### Schwann Cell Development

Schwann cells are varied group of glial cells that produce protective myelin sheaths and support NMJ function, remodeling, and regeneration (Son et al., 1996). SCs undergo three main transitions during development: (1) from migrating neural crest cells (NCCs) to SC precursors (SCPs), (2) SCPs to immature SCs, and (3) immature SCs to a mature myelinating or non-myelinating SCs (Jessen and Mirsky, 2005). These transitions and SC survival are dependent upon morphogens secreted from axons with which SCPs and SCs continuously associate (Jessen and Mirsky, 1999). However, the molecular mechanisms that regulate neural crest cell differentiation into SCs have not been fully elucidated.

Early in the process of neurulation, dorsally located NCCs segregate from the neural tube and migrate in ventrally (Jessen and Mirsky, 2005). The basic helix-loop-helix (bHLH) transcription factor Sox10 is expressed early by all NCCs (Kuhlbrodt et al., 1998; Woodhoo and Sommer, 2008). While high expression of Sox10 persists in glial and melanocyte NCC derivatives of the peripheral nervous system (PNS), its expression is downregulated in other NCC derivatives (Kuhlbrodt et al., 1998; Woodhoo and Sommer, 2008). The continued high expression of Sox10 is dependent on the expression of Pax3 (Kioussi et al., 1995; Blanchard et al., 1996; Doddrell et al., 2012), which is regulated in part by histone deacetylases 1 and 2 (HDAC1/2) (Jacob et al., 2011). Together, Sox10 and Pax3 induce the expression of key SC lineage genes including fatty acid binding protein 7 (Fabp7) and myelin protein zero (MP0) (Kioussi et al., 1995; Blanchard et al., 1996; Doddrell et al., 2012).

While Sox10 is necessary for SC specification, it is not sufficient. In the developing PNS, SCPs migrate alongside MN axons extending to targeted regions of innervation (Jessen and Mirsky, 1999). Moreover, migrating SCPs are dependent upon signals from these axons, such as Neuregulin-1 (NRG1), for appropriate development and survival (Jessen and Mirsky, 1999). In NCC cultures, NRG1 suppresses neuronal differentiation and promotes glial specification (Shah et al., 1994). NRG1 binds ErbB2/3, an obligate heteromeric receptor tyrosine kinase pair, on SCPs to activate key downstream signal transduction cascades that are essential for both proliferation and directed migration (Newbern and Birchmeier, 2010).

Immature SCs develop after SCPs cease migration and populate axons that are still projecting to their targeted region of innervation, while acquiring a set of properties that clearly distinguish them from SCPs. Specifically, they cease migration, become dependent on autocrine signaling for survival, and deposit an organized basal lamina (Jessen and Mirsky, 2005). Additionally, increased notch signaling is a critical mediator of the SCP transition to immature SCs, with a loss of notch signaling preventing immature SC formation (Woodhoo et al., 2009).

### Generation of Schwann Cells From Pluripotent Stem Cells

Methods to differentiate hiPSCs into SCs have mimicked developmental process by first generating neural crest-derived SC precursors from hiPSCs. Neural crest stem cells have been derived from hiPSCs via FACS selection of p75+ cells derived from EBs cultured in stromal-cell-conditioned media, FGF2, and B-27 supplement (Liu et al., 2012). These cells were subsequently differentiated into a nearly pure population of SCs expressing glial fibrillary acidic protein, S100, and p75 through culture in mesenchymal stem cell medium supplemented with Neuregulin-1 for 40 days. A similar method using EB formation and FGF2 treatment was later developed that shortened the time of induction to 6 days (Huang et al., 2017).

To overcome the challenges with low reproducibility and throughput in EB cultures, methods for directed differentiation of hiPSCs to SCs have been developed. Specifically, sequential treatment of naïve hiPSCs with TGF-β and GSK-3β inhibitors followed by NRG1 produced SC precursors in 18 days (Kim et al., 2017). These precursors were further differentiated through treatment with NRG1, retinoic acid, platelet-derived growth factor-BB (PDGF-BB), and forskolin into SCs. This method shortened the total differentiation time from approximately 41 days (Huang et al., 2017) to approximately 32 days (Kim et al., 2017). However, a more recent protocol has been developed for the derivation of direct Schwann-cell precursors (SCPs) from SOX10-reporting hiPSCs that only required a total differentiation time of 21 days and allowed for *in vitro* culture up to 80 days with maintained expression of the SC proteins S100b, glial fibrillary acidic protein (GFAP), and galactosylceramidase (Mukherjee-Clavin et al., 2019). With all these methods, differentiated SCs show increased expression of SC-specific markers such as GFAP and S100β.

In addition to protein expression, hiPSC-derived SCs have been studied for their secretion rates of neurotrophic factors (Huang et al., 2017; Kim et al., 2017), ability to myelinate primary neurons (Liu et al., 2012; Kim et al., 2017), and ability to accelerate nerve healing within rodent sciatic nerve injury models (Huang et al., 2017; Kim et al., 2017). hiPSC-derived SCs have additionally been used in coculture with hiPSC-derived neurons to allow for stronger neuronal outgrowth within a 3D tissue-engineered skin model (Muller et al., 2018). Recent studies have further confirmed critical physiological roles of SCs in the stabilization and maintenance of NMJs *in vitro* (Singh and Vazquez, 2019; Martins et al., 2020), forming a foundation for the future development of novel biomimetic NMD models.

### Neuromuscular Junction Development

**FIGURE 3:**
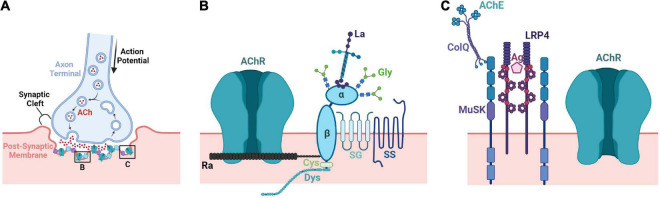
Structural and molecular architecture of the neuromuscular junction. **(A)** The NMJ is comprised of three components: (1) the axonal terminal of an MN (pre-synapse), (2) the basal lamina of the synapse (synaptic cleft), and (3) the sarcolemma (membrane) of a muscle fiber (post-synapse). Following the conduction of an action potential to the axon terminal, Ca^2+^ influx occurs at the presynaptic terminal releasing ACh-containing vesicles into the synaptic cleft. Released ACh can then bind to AChRs on the sarcolemma creating an endplate potential and eventually muscle contraction. **(B)** AChE secreted by the muscle binds to ColQ and inactivates residual ACh within the synapse. ColQ binds to MuSK to help stabilize the synapse. The synaptogenic proteoglycan agrin secreted by MNs binds to LRP4 to facilitate formation of the NMJ. **(C)** AChRs are stabilized by dystrophin-associated glycan (DAG) complexes. The AChR-clustering protein rapsyn connects AChRs to the DAG complex and dystrophin anchors the complex to the SkM cytoskeleton. Lamins and glycans additionally connect the complex to the ECM while the sarcoglycan and sarcospan stabilize the DAG complex within the membrane. ACh, acetylcholine; AChE, acetylcholine esterase; ColQ, collagen Q; MuSK, muscle-specific tyrosine kinase receptor; Ag, agrin; LRP4, low density lipoprotein receptor 4; AChR, acetylcholine receptor; AChR, acetylcholine receptor; La, laminin; α/β, α/β dystroglycan; Gly, glycans; SG, sarcoglycan; SS, sarcospan; Ra, rapsyn; Cys, cysteine; Dys, dystrophin.

The NMJ is a chemical synapse formed between MNs and SkM that allows the transmission of motor commands from the CNS (Figure 3). MNs communicate with SkM through the release of acetylcholine (ACh) into the synaptic cleft of the NMJ. ACh receptors (AChRs) located on muscle fibers are activated and depolarize the muscle cell which triggers calcium release from the sarcoplasmic reticulum initiating a contraction (Fambrough, 1979). Reciprocal signaling between MNs and SkM is important for the formation and maintenance of NMJs as highlighted by the coordination required for complex movements and sensory-motor feedback.

During development, immature SCs migrate with MNs toward the periphery (Sugiura and Lin, 2011) and differentiate into either axonal SCs that myelinate axon extensions or terminal SCs that support the NMJ formation. Terminal SCs proliferate extensively around the NMJ (Hirata et al., 1997) and then cover or “cap” the nerve terminal with their processes (Court et al., 2008). Muscular innervation is preceded by the localization of small aneural AChR clusters to the central region of muscle fibers in a process called prepatterning. During this process, the MN terminal releases agrin which binds to the muscle-specific kinase (MuSK) co-receptor, low-density lipoprotein receptor related protein 4 (LRP4), promoting activation and transphosphorylation of MuSK. SC processes contact pre-patterned AChR clusters prior to the nerve and cover more of the postsynaptic membrane than axonal terminals during early synapse formation (Flanagan-Steet et al., 2005). Additionally, SCs express active agrin and encourage aggregation of AChRs on muscle fibers (Yang et al., 2001). In mice, SC loss results in MN defasciculation, but MNs still project toward muscle targets implying that SCs are not required for initial nerve–muscle contacts (Woldeyesus et al., 1999; Lin et al., 2000). However, further growth and maintenance of this early synapses is halted in SC absence (Riethmacher et al., 1997), suggesting that SCs are critical for NMJ homeostasis.

Eventually, innervation of muscle fibers induces the formation of larger, neural AChR clusters forming stable NMJs in the middle region of muscle fibers. Mice with a mutated agrin gene (*agrin^–/–^*) are unable to form NMJs; however, these mice can form aneural AChR clusters on muscle fibers prior to innervation (Lin et al., 2001). Conversely, aneural clusters are not formed in *MuSK^–/–^* mice, and their muscle fibers demonstrate a uniform distribution of AChRs with a broader region of innervation containing highly branched MN terminals. Neuronal agrin does not induce AChR clusters in *MuSK^–/–^* muscle cells (Glass et al., 1996); however, agrin sensitivity can be restored through expression of wild-type MuSK (Zhou et al., 1999). Interestingly, synapse formation can be rescued in *agrin^–/–^* mice with ectopic MuSK expression (Kim and Burden, 2008). Together, this suggests the importance of MuSK for aneural AChR clustering and prepatterning prior to innervation while agrin is also needed for neural AChR clustering and NMJ formation. Additionally, SC processes influence nerve terminal growth and are required for both the formation and maintenance of developing NMJs (Reddy et al., 2003).

Neuromuscular junction formation is also influenced by several extracellular components. For example, MuSK has a cysteine-rich domain (CRD) that shares homology with the WNT receptor, Frizzled. As a result, WNT proteins bind and activate MuSK prior to innervation, when neural agrin is absent (Barik et al., 2016). This signaling can regulate axon guidance as well as induce aneural cluster formation (Li et al., 2018). Moreover, both canonical and non-canonical WNT pathways are affected in transgenic mice with MuSK CRD deletions (Messeant et al., 2017). Many components of the extracellular matrix (ECM) have important regulatory roles in myogenesis and synaptogenesis. Within the synaptic basal lamina, ECM molecules help guide the process of innervation and are crucial to formation of post-synaptic density as well as organization and maintenance of functional appositions of the pre- and post-synaptic elements. The dystrophin-associated glycoprotein complex (DGC), through its α-dystroglycan subunit, organizes a functional scaffold in the basal lamina including perlecan, acetylcholinesterase/ColQ, and laminin that stabilizes AChR clusters (Jacobson et al., 2001). The DGC additionally connects networks of laminins and collagens to one another by nidogen and anchors them to the sarcolemma through the sarcoglycan-sarcospan subcomplex and intracellular cytoskeleton through dystrophin (Fox et al., 1991; Jacobson et al., 2001). The laminin β2 chain plays a role in synapse maturation by binding and clustering voltage-dependent calcium channels (VDCC) in the active zone of the NMJ. A reduced number of active zones and pre-synaptic release of ACh is observed in mice lacking laminin β2 (Rogers and Nishimune, 2017). Mice lacking ColQ, collagen XIII, collagen IV, or collagen VI also exhibit immature nerve terminals and/or NMJs (Sigoillot et al., 2016; Cescon et al., 2018; Zainul et al., 2018).

After forming functional NMJs with their target, MNs impinge on muscle fiber structural and functional diversity. A single muscle is composed of several fiber types that are innervated by specific classes of MNs. MNs are subdivided into three groups based on the type of muscle fiber they innervate: (1) alpha MNs, which innervate force generating extrafusal fibers, (2) gamma MNs, which innervate the proprioceptive intrafusal fibers, and (3) beta MNs, which innervate both extrafusal and intrafusal fibers. Alpha MNs are the most abundant of these classes and are categorized as SFR (slow-twitch, fatigue-resistant), FFR (fast-twitch, fatigue-resistant), and FF (fast-twitch, fatigable) reflecting the type of extrafusal muscle fiber they innervate (Totosy de Zepetnek et al., 1992). MNs are intrinsically competent to recognize and connect to either fast or slow muscle fibers (Landmesser, 2001). Slow MNs start to specifically express the synaptic vesicle glycoprotein 2a (SV2A) (Chakkalakal et al., 2010) as well as the estrogen-related receptor beta (ESRRB) (Enjin et al., 2010) soon after birth. Conversely, fast MNs specifically express the calcitonin-related polypeptide alpha (CALCA) and the chondrolectin (CHODL) (Enjin et al., 2010). The Notch ligand delta-like homolog1 (DLK1) has also been identified as a necessary regulator of fast MNs (Muller et al., 2014). Understanding of the influence of innervation by specific MN types upon SkM phenotype could provide important insight into certain NMDs that preferentially target specific muscle fiber types.

## Current Models for Studies of Neuromuscular Junction Function and Disease

### Animal Models

Various animal models have been broadly utilized to advance our understanding of the formation, function, and malfunction of NMJs during the development and progression of NMDs. Moreover, they have been used to study the pathophysiology and develop pharmacotherapies for NMDs. Specifically, *Caenorhabditis elegans* (Sleigh and Sattelle, 2010), zebrafish (Babin et al., 2014), *Drosophila* (Shields et al., 2017), and mice (Hsieh-Li et al., 2000) have been extensively employed to investigate the precise anatomy and function of NMJs. Their ease of genetic manipulation, tractable anatomy, relatively rapid growth, and low cost have contributed to their extensive use (Dawson et al., 2018). The mouse NMJ has been particularly useful due to its large size and accessibility, facilitating microscopic studies by immunofluorescence histology and functional analyses by electrophysiology (Webster, 2018). The imaging studies, in particular, have enabled enhanced understanding of how localization and density of pre-synaptic, post-synaptic, and synaptic proteins are rearranged or lost in disease states.

However, experimental results in animal models may have limited translational value due to distinct anatomical differences between animal and human NMJs. For example, the murine NMJ and human NMJ exhibit substantially different proteomes and the larger, more pretzeled murine NMJ readily remodels with age whereas the smaller, more fragmented human NMJ is mostly conserved (Jones et al., 2017). Interestingly, the density of the active portion of the human NMJ is greater compared to its mouse counterpart (Jones et al., 2017). Additionally, disease phenotypes in animals can vary widely from those in humans in terms of progression, severity, and etiology (Vainzof et al., 2008). High levels of inbreeding limit genetic diversity within common animal models and controlled environments prevent genetic drift, while removing common viral and microbial agents that can influence human NMD pathogenesis (Dawson et al., 2018). The inability of animals to fully capture the genotypic heterogeneity and allelic variations observed across human individuals has hindered the clinical success of NMD drugs validated through animal models (Vainzof et al., 2008). Only a small fraction of drugs that enter clinical trials are approved as many result in unanticipated drug responses and toxicities (Vainzof et al., 2008). This situation has prompted development of *in vitro* human models of NMJ and NMDs that could allow studies of disease and pharmacological effects in a personalized and clinically more relevant fashion.

### Two-Dimensional *in vitro* Models

**FIGURE 4:**
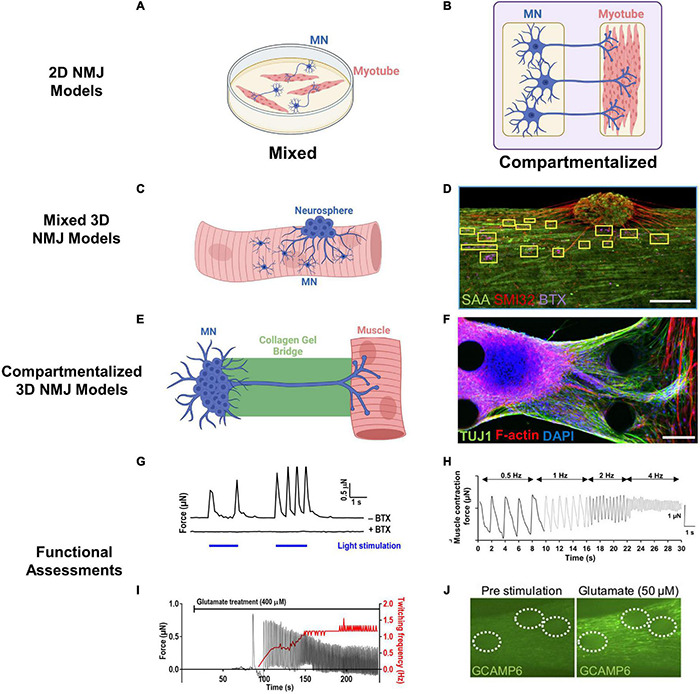
Engineered NMJ models. **(A,B)** Schematics of 2D NMJ co-culture models in mixed **(A)** and compartmentalized **(B)** configuration. **(C)** Mixed 3D NMJ co-culture systems incorporate MNs or neurospheres into SkM during 3D tissue formation. **(D)** Representative mixed 3D NMJ model with immunofluorescent staining of muscle sarcomeres (SAA), neurite extensions (SMI32), and acetylcholine receptors (BTX). Scale bar, 200 μm (Bakooshli et al., 2019; Copyright 2019, *eLIFE*). **(E)** Compartmentalized 3D NMJ co-culture systems culture MNs and SkM in separate compartments bridged by an extracellular matrix gel to facilitate axonal outreach and SkM innervation. **(F)** Representative compartmentalized NMJ model with immunofluorescent staining of neurite extensions (TUJ1) and myotubes [filamentous (F)-actin]. Scale bars, 100 μm (Osaki et al., 2018; Copyright 2018, *Science Advances*). **(G)** Example of optogenetic control of 3D NMJ models through blue light illumination (blue bars) of ChR2^*H134R*^-HBG3-MN neurospheres inducing contraction in muscle (*y*-axis) as measured by pillar displacement within a microfluidic system. Administration of α-bungarotoxin (BTX) prevented MN-induced contractions (Uzel et al., 2016; Copyright 2016, *Science Advances*). **(H)** Example of muscle contraction induced by electrical stimulation of MNs at varying frequencies (0.5–4 Hz) (Osaki et al., 2018; Copyright 2018, *Science Advances*). **(I)** Representative recording of contractile force (*y*-axis) in 3D SkM-MN co-culture induced by glutamate stimulation of neurospheres (Uzel et al., 2016; Copyright 2016, *Science Advances*). **(J)** Representative recording of glutamate-induced Ca^2+^ transients in 3D SkM-MN co-culture with muscle-specific expression of GCaMP6 reporter. MN neurospheres are encircled by dashed lines (Bakooshli et al., 2019; Copyright 2019, *eLIFE*).

When developing *in vitro* models of NMJ, it is important to both consider its anatomical structure and enable relevant biological and functional studies. Initial rodent models of NMJ entailed mixed 2D co-cultures of myotubes with dissociated MNs (Kengaku et al., 1991) or spinal cord explants (Askanas et al., 1987), either plated simultaneously or sequentially (Figure 4A). Axonal projections in these co-cultures extended from the MNs to form NMJs with the myotubes that in turn exhibited functional post-synaptic potentials. Development of *in vitro* human models of the NMJ (Guo et al., 2011; Demestre et al., 2015; Yoshida et al., 2015) have additionally opened doors to personalized modeling of NMDs. For example, hiPSCs from spinal muscular atrophy (SMA) patients exhibited impaired AChR clustering which was ameliorated with valproic acid and antisense oligonucleotide treatment (Yoshida et al., 2015). The main advantages of these 2D culture models were relative simplicity and use of a flat substrate allowing for efficient and direct analysis of cell morphology and pathological features.

However, AChR clustering in mixed 2D co-cultures exhibits poor co-localization of pre- and post-synaptic structures compared to native NMJs, hindering the ability to recapitulate the intricacies of specific NMDs (Das et al., 2010; Umbach et al., 2012). Specifically, without proper spatial cues, myoblasts in mixed 2D cultures fuse into randomly oriented and branched myotubes limiting formation of elongated myofibrils and mature sarcomeres (Bettadapur et al., 2016). These myotubes will often delaminate after few days of culture as they start to generate more mechanical stress against the underlying substrate, thus not providing sufficient time for proper NMJ maturation (Wang et al., 2012; Sun et al., 2013). Anatomical considerations also hinder physiological relevance of these models. *In situ*, the soma of the MNs reside in the spinal cord with only the axons projecting and physically interacting with the myofibers. Plating MNs on top of myotubes in mixed 2D co-cultures is therefore anatomically incorrect and may alter the physiology of one or both cell types. Additionally, measuring SkM force generation is not possible on most conventional culture substrates and individual analysis of each cell type, both functional and molecular, is often impractical.

To overcome these technical challenges, researchers have developed several types of tunable culture surfaces and microfabricated devices to engineer more biomimetic NMJs with improved anatomical organization. Use of topographical cues, such as polylactic acid (PLA) or polycaprolactone (PCL) nanofibers, served to align murine myoblasts and improve NMJ formation with co-cultured rat embryonic spinal MNs (Luo et al., 2018; Das et al., 2020). Micropatterning of alternating soft and stiff extracellular matrix strips increased expression of the NMJ markers MuSK and LRP4, improved myoblast fusion, and augmented AChR cluster size when rat primary MNs were co-cultured with both human and mouse SkM cells (Happe et al., 2017). Micropatterning techniques have also been shown to promote hiPSC-derived MN survival (Burbulla et al., 2016) and align human myoblasts (Ebrahimi et al., 2018).

Furthermore, incorporation of SCs has improved viability and survival of MNs in long-term 2D cultures *in vitro* while supporting longer, myelin ensheathed axonal projections in rodent models *in vivo* (Haastert et al., 2005; Honkanen et al., 2007; Paivalainen et al., 2008; Viader et al., 2011; Hyung et al., 2015). Within human cell lines, increased myotube number, length, and viability were observed in both SC/SkM cocultures and SC/SkM/MN tricultures highlighting the synergistic relationships among these cell types (Singh and Vazquez, 2019). Additionally, self-organization of hiPSC-derived NMJs has been accomplished following simultaneous generation of MNs, SkM, and SCs from a bipotent NMP population (Lin et al., 2019) fated to form both spinal neuroectodermal and associated musculoskeletal mesodermal cell derivatives (Gouti et al., 2017). Within this system, contractile and electrophysiological activity driven by functional NMJs was supported by the presence of terminal SCs and myelinated axons.

Additionally, development of compartmentalized 2D NMJ models has allowed for MN somas and myotubes to be spatially separated increasing the biomimetic nature of these co-cultures (Figure 4B). In the first example of a compartmentalized NMJ model, neurons derived from murine embryonic stem cells and fused C2C12 myoblasts were cultured separately in a microfluidic device and connected only through axon extensions (Park et al., 2013). Similar compartmentalized 2D models have been used to study rodent synaptic formation (Tong et al., 2014), AChR clustering (Southam et al., 2013), and MN-induced calcium transients in myotubes (Ionescu et al., 2016). A compartmentalized 2D NMJ model between primary embryonic rat MNs and myotubes inside an automated device allowed measurements of MN-initiated muscle contractile force through cantilever displacement (Smith et al., 2013). Similar studies testing effects of bungarotoxin, BOTOX^®^, and curare were performed in a human co-culture platform where video recording analysis was used to measure amplitude and frequency of MN-induced myotube contractions (Santhanam et al., 2018). Additionally, lentiviral transduction of human MNs with channelrhodopsins has enabled a more precise, light-mediated control over MN activity in NMJ co-cultures (Steinbeck et al., 2016). Use of single-donor hiPSC-derived SkM cells and MNs has further enabled patient-specific disease modeling (Guo et al., 2020a) with capability to assess NMJ function in response to electrical stimulation of MNs. While these 2D compartmentalized platforms partly recapitulate *in vivo* organization through physical separation of MNs and SkM cells, they lack the structural 3D complexity of the native innervated muscle.

### Three-Dimensional *in vitro* Models

The lack of the 3D cell–cell and cell–ECM interactions in 2D NMJ models has prompted the development of 3D *in vitro* models of innervated SkM (Figures 4C–F), which despite being more expensive, time-consuming, and lower throughput than 2D cultures, are expected to provide a more physiologically relevant platform for NMD studies. The first example of a mixed 3D NMJ model incorporated fetal rodent nerve explants within 3D SkM constructs resulting in the formation of functional NMJs and expression of more mature myosin heavy chain (MHC) isoforms (Larkin et al., 2006). A similar mixing technique using neonatal rat myoblasts and embryonic ventral horn neurons improved myotube cytoskeletal organization and augmented force production of engineered SkM (Martin et al., 2015). Direct co-culture of mouse-derived MN spheroids and SkM allowed for contraction following glutamic acid activation of MNs that could be inhibited by curare treatment, a NMJ antagonist (Morimoto et al., 2013). However, compared to native NMJs, AChRs clustering remained relatively diffuse in these systems (Morimoto et al., 2013). These first-generation 3D NMJ models demonstrated the utility of 3D platforms to generate functional NMJs but their non-compartmentalize nature and use of embryonic rodent cells limit their utility for studies of human NMDs.

Consequently, recent efforts have focused on the development of human 3D NMJ co-cultures. For example, addition of hiPSC-MN clusters into 3D SkM/hydrogel suspensions or to pre-formed SkM tissues allowed generation of mixed 3D NMJ models (Figures 4C,D) where consequences of functional connectivity between MNs and muscle fibers were studied by recording calcium transients or contractile force generation (Osaki et al., 2018; Bakooshli et al., 2019; Rimington et al., 2021). Compared to 2D monolayers, 3D MN spheroid co-culture with SkM increased axon length and expression of SMI32, a marker of MN maturity (Rimington et al., 2021), while the presence of MNs improved the overall structure and function of myotubes (Bakooshli et al., 2019; Rimington et al., 2021), revealing the mutually beneficial effects of MNs and SkM within 3D co-culture systems. Interestingly, functional innervation was achieved following 2 weeks of culture within 3D, but not in comparable 2D NMJ co-cultures, and expression of the mature AChR ε-subunit was observed only in 3D NMJ co-cultures (Bakooshli et al., 2019). Beyond mixed 3D NMJ models, compartmentalized microdevices (Figures 4E,F) have been developed to spatially separate MN spheroids and engineered SkM and connect them via axon-permissive channels to more appropriately mimic *in vivo* muscle innervation (Uzel et al., 2016; Osaki et al., 2018; Vila et al., 2021). Through this compartmentalization, visualization of 3D neurite outgrowth and engineered SkM innervation is greatly simplified, similar to studies in 2D compartmentalized co-cultures.

Incorporation of SCs can further improve longevity and biomimetic organization of 3D NMJ models as shown in rodent co-cultures, where MN–SC interactions led to extended, myelinated axonal projections of MNs with improved viability (Gingras et al., 2008; Hyung et al., 2021), while optical stimulation of murine MNs acted reciprocally on SCs to enhance the myelination process, leading to the formation of thicker myelin sheaths (Hyung et al., 2019). Similarly, in human organoids, NMJs identified by accumulation of αBTX clusters in muscle fibers encompassed both myelinated axons and capping terminal SCs and were shown to be functional by curare-induced block of muscle activity (Faustino Martins et al., 2020).

Motor neuron activation within 3D NMJ models has been achieved through addition of the neurotransmitter glutamate (Osaki et al., 2018; Bakooshli et al., 2019) or its mimic *N*-Methyl-D-aspartate (NMDA), optogenetic control (Osaki et al., 2018; Vila et al., 2019, 2021), or direct electrical stimulation (Osaki et al., 2018; Rimington et al., 2021). Glutamate stimulates MNs (Figure 4I) through binding to α-amino-3-hydroxy-5-methyl-4-isoxazolepropionic (AMPA), kainic acid (KA), and NMDA receptors while NMDA specifically targets NMDA receptors (Newcomer et al., 2000). High doses of glutamate or NMDA can be used to study excitotoxicity (over-activation of glutamate receptors) while lower doses can access NMJ model sensitivity (Liu et al., 2007). Optogenetic control in NMJ models (Figure 4G) relies on genetic modification of MNs to express light-sensitive channels, such as channelrhodopsin, that induce an action potential and subsequent muscle contraction in response to blue light. This method, though utilizing genetically altered MNs, allows for spatiotemporal and noninvasive control over motor units. Direct electrical stimulation, while impractical in mixed co-culture systems as it would excite both MNs and SkM cells, can be implemented in compartmentalized systems to stimulate MNs (Figure 4H).

To assess NMJ functionality, recordings of calcium transients have been used as an indicator of MN-induced muscle excitation and gCaMP6 (Bakooshli et al., 2019), a genetically encoded calcium indicator, has been used to visualize calcium flow through muscles (Figure 4J). Furthermore, MN-innervated engineered SkM tissues can be cultured on microfabricated pillars, displacement of which can be imaged to assess muscle contractions induced via glutamate or light-stimulated MN activity (Uzel et al., 2016; Vila et al., 2019, 2021; Afshar et al., 2020). In addition to indirect functional measurements by video recordings, contractile force generation in mixed 3D NMJ co-cultures can be directly measured by a force transducer (Martin et al., 2015; Rizzuto et al., 2017; Rimington et al., 2021), which allows for assessment of the muscle force-length relationship and could be used for detailed functional studies in compartmentalized 3D NMJ models, similar to those performed in native nerve-muscle preparations (Martin et al., 2015; Rizzuto et al., 2017). Finally, transfer of MN activity to SkM can be blocked through a variety of AChR inhibitors including α-bungarotoxin (Osaki et al., 2018; Vila et al., 2019) and tubocurarine (Bakooshli et al., 2019; Rimington et al., 2021) to further validate NMJ functionality.

## Neuromuscular Diseases

**FIGURE 5:**
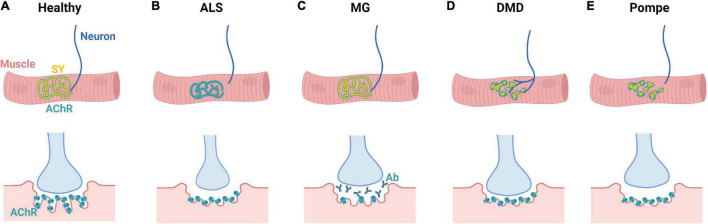
NMD-specific changes in NMJ morphology. **(A)** Mature human NMJs present with a characteristic “pretzel” shape and contain both synaptophysin (SY) and acetylcholine receptors (AChRs). NMJ endplates exhibit distinct membrane folds that extend into the muscle cell cytoplasm. **(B)** In ALS, marked decrease in synaptophysin and NMJ size occurs alongside denervation. Additionally, ALS patients present with smaller nerve terminals and flattened synaptic clefts. **(C)** In MG, NMJ morphology is generally maintained; however, there is synaptic accumulation of autoantibodies (Ab), decrease in AChRs, and shortening of synaptic clefts. **(D)** In DMD, there is marked axonal branching and NMJ fragmentation alongside shortening of the synaptic cleft. **(E)** In Pompe disease, there is denervation alongside NMJ fragmentation and increased rates of MN apoptosis.

Neuromuscular diseases originate from various pathophysiological mechanisms, exhibit diverse symptoms, and differentially affect the NMJ (Figure 5). As such, they have historically been divided into subcategories and viewed through either a neurogenic or myogenic lens. However, increasing evidence for the important roles of cellular crosstalk in NMD pathogenesis suggest that modeling of the entire motor unit is necessary for proper studies of NMDs. In the following section, we present five NMD examples with diverse causes and manifestations highlighting the pathogenic roles of both MNs and SkM. First, we discuss ALS, a genetic disorder viewed to primarily affect MNs. Second, we consider MG, an autoimmune disorder focused upon the NMJ. Third, we examine DMD, a muscular dystrophy resulting from loss or truncation of the sarcolemmal protein dystrophin. Fourth, we review DM, a muscular dystrophy arising from toxic RNA repeats. Fifth, we discuss Pompe disease, a glycogen storage disorder affecting multiple cell types. For each NMD, we highlight most representative *in vitro* (Table 1) and *in vivo* models and offer perspective on future progress needed to advance NMD modeling toward translational applications.

**TABLE 1:** Selected *in vitro* human models of NMD.

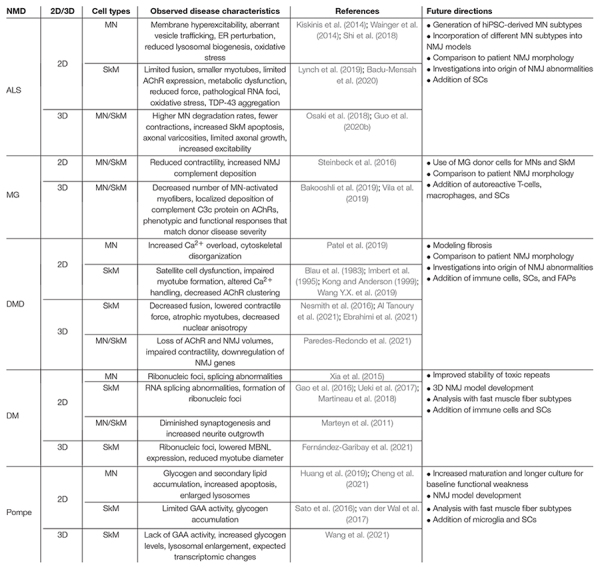

### Amyotrophic Lateral Sclerosis

Amyotrophic lateral sclerosis is late-onset, progressive NMD caused by SkM and MN wasting resulting in paralysis, respiratory failure, and death (Brown and Al-Chalabi, 2017). It is characterized by muscle stiffness and spasticity, but many patients also exhibit cognitive and behavioral changes (Oskarsson et al., 2018). Unfortunately, no ALS-specific biomarkers are currently known resulting in lengthy diagnosis periods and delayed treatments (Oskarsson et al., 2018). While no curative therapy is currently available, approved drugs, such as Edaravone and Riluzole, limit disease progression and may lengthen patient survival up to several months (Jaiswal, 2019). Approximately 90–95% of patients have sporadic ALS and 5–10% have familial disease, with no clear clinical or pathological differences between the groups (Loeffler et al., 2016). Over 100 genes have been attributed to familial ALS with the most commonly affected genes being *C9ORF72*, *SOD1*, *TARDBP*, and *FUS*, typically in combination (Wroe et al., 2008). The precise molecular mechanisms of ALS are unknown; however, many contributing factors have been proposed including protein aggregation (Ross and Poirier, 2004), excitotoxicity (Rothstein, 1995), aberrant nucleocytoplasmic or endosomal transport (Zhang et al., 2015), dysfunctional RNA metabolism (Strong, 2010), oxidative stress (Barber and Shaw, 2010), and axonal deformations (Bilsland et al., 2010). Although MN degradation is characteristic of ALS, denervation of the NMJ occurs first (Tremblay et al., 2017). Interestingly, ALS exhibits a preferential degradation of MNs with early loss of fast-fatigable MNs followed by fast fatigue-resistant, and then slow MNs (Tremblay et al., 2017). NMJs in ALS patients exhibit endplate fragmentation (Bjornskov et al., 1975), smaller endplates and nerve terminals (Tsujihata et al., 1984), flattened synaptic clefts (Yoshihara et al., 1998), and reduced mitochondrial presence within the nerve terminal (Tsujihata et al., 1984). Some small nerve terminals have been observed over distorted endplates, suggesting the possibility for NMJ regeneration (Yoshihara et al., 1998).

While ALS research has primarily focused upon MN pathology, studies with animal models have underlined the importance of pre-symptomatic SkM changes including atrophy and denervation (Loeffler et al., 2016). Prior to disease onset in *SOD1* mice, there is an upregulation in muscle developmental genes (De Oliveira et al., 2014), a decrease in CDK5 (myogenic marker) (Park and Vincent, 2008), sarcoplasmic accumulation of neuronal NOS (Suzuki et al., 2010), and a loss in muscle volume (Kraft et al., 2007). Muscle-specific overexpression of *SOD1* in mice has caused oxidative stress and muscular wasting without motor defects (Dobrowolny et al., 2008), while neuron-specific expression of *SOD1* did not result in neuron abnormalities (Lino et al., 2002). Satellite cells from pre-symptomatic mice exhibit upregulated Pax7 expression (Manzano et al., 2011) and reduced proliferative capacity (Manzano et al., 2013). ALS patients exhibit a similar dysfunction in satellite cell proliferation (Scaramozza et al., 2014), indicating intrinsic muscle pathology in ALS as satellite cells are not directly innervated. Additionally, electrophysiological postsynaptic alterations presented in *SOD1* mice prior to 6 weeks of age (Rocha et al., 2013) and decreased expression of choline acetyltransferase (ChAT) and vesicular acetylcholine transporter resulted in cholinergic dysfunction prior to MN degradation (Casas et al., 2016). In these mice, early NMJ dysfunction is accompanied by Ca^2+^ and reactive oxygen species accumulation, mitochondrial failure, and impaired transport within axons (Fischer-Hayes et al., 2013; Pollari et al., 2014). Clinically, pathophysiological axonal excitability is more pronounced within distal axonal branches (Nakata et al., 2006) and muscle denervation occurs before spinal cord MN degradation (Pollari et al., 2014). Based upon these observations, the “dying back” hypothesis of ALS suggests that this disease progresses through a retrograde degeneration of MNs from the periphery (Moloney et al., 2014) contrasting with the “dying forward” hypothesis that suggests glutamate excitotoxicity from cortical MNs advances forward to the periphery (Eisen et al., 1992). Additionally, astrocytes express most ALS related genes, and their dysregulation leads to neuroinflammation, oxidative stress, excitotoxicity, and protein aggregation further supporting the non-cell autonomous nature of ALS (Halpern et al., 2019).

Several animal models of ALS have been developed with a variety of mutations. Transgenic mice expressing mutant human *SOD1* have been heavily studied and particularly useful in understanding pathophysiology of ALS. These mice progressively accumulate *SOD1* within their muscles causing endoplasmic reticulum stress (Chen et al., 2015), recapitulate characteristic degradation of MNs and paralysis, and importantly demonstrate the non-cell autonomous nature of ALS (Nagai et al., 2007). Additionally, these mice exhibit altered gene expression related to muscle repair (De Oliveira et al., 2014), reduction in muscle volume (Marcuzzo et al., 2011), decreased proliferative capacity of satellite cells (Manzano et al., 2013), and a slow fiber-type shift (Hegedus et al., 2007). Studies with *SOD1* mice have been useful to identify potential treatments with glial cell line-derived neurotrophic factor (GDNF) to rescue MN function through overexpression within the SkM (Li et al., 2007), stem cell-based delivery (Suzuki et al., 2007), and intramuscular injection of GDNF (Suzuki et al., 2008). Unfortunately, overexpression of healthy human *SOD1* in these mice results in axonopathy undermining mutational importance within this disease model (Joyce et al., 2011) and spontaneous copy number deletions limit disease severity increasing variability within *SOD1* mouse studies (Zwiegers et al., 2014). Additionally, rodent astrocytes exhibit significantly varied expression of many ALS-related genes, are less structurally and functionally diverse, and express a 10-fold decrease in glial fibrillary acidic protein-positive processes than human astrocytes, further limiting translational relevance of this model when studying the role of astrocytes in ALS (Oberheim et al., 2009).

To specifically study the effect of ALS on MN populations, hiPSC-based platforms have been widely employed. The first example of drug screening in ALS hiPSC-derived MNs used cells from patients with *TARDBP* mutations that exhibited decreased neurite length, which allowed identification of anacardic acid (a histone acetyltransferase inhibitor) as a potential ALS therapeutic agent (Egawa et al., 2012). These platforms have been expanded to the other ALS-associated mutations and have included assessments of membrane hyperexcitability (Wainger et al., 2014), vesicle trafficking (Shi et al., 2018), ER perturbation (Kiskinis et al., 2014), lysosomal biogenesis (Shi et al., 2018), and oxidative stress (Kiskinis et al., 2014). To model sporadic ALS in hiPSC-MNs, several models were combined to recapitulate heterogeneous neuronal degeneration, protein aggregation, and cell death and identify ropinirole as a potential therapeutic candidate (Fujimori et al., 2018). However, these models utilize rather immature neurons to model a disease that presents in mid to late life and only consider cell-autonomous effects of ALS. Accelerated aging through molecular manipulation, such as progerin overexpression (Miller et al., 2013), may improve the clinical relevance of these models.

To investigate non-cell autonomous contributions to ALS in a cell-specific manner, hiPSC-derived SkM and astrocyte models of ALS have been developed. Initial hiPSC-derived ALS SkM cells induced through both MyoD overexpression (Lenzi et al., 2016) and small molecule differentiation (Swartz et al., 2016) exhibited typical maturation patterns with limited pathologic alterations. Recently, however, a SkM model derived from *SOD1*-mutant ALS patient hiPSCs exhibited delayed and lower rates of fusion, smaller myotube size, limited AChR expression, metabolic dysfunction, and significantly reduced force production compared to healthy cells (Badu-Mensah et al., 2020). Additionally, a model using hiPSC-derived myotubes from *C9ORF72* mutant patients exhibited pathological RNA foci, dipeptide repeat proteins, oxidative stress, and TDP-43 aggregation (Lynch et al., 2019). Beyond cultured hiPSC-SkM cells, hiPSC-derived astrocytes from ALS patients exhibit decreased expression of LC3-II causing p62 accumulation and modulated autophagy in HEK293T cells treated with astrocyte conditioned media (Madill et al., 2017). Additionally, *C9ORF72* mutant hiPSC-derived astrocytes exhibited pathological RNA foci and dipeptide repeat proteins while causing MNs to undergo progressive action potential loss upon co-culture (Zhao et al., 2020). Through CRISPR-based removal of the *C9ORF72* repeat, these phenotypes were reverted indicating both cell-autonomous astrocyte pathology and non-cell autonomous MN pathophysiology attributed to astrocytes (Zhao et al., 2020). Metabolically, *C9ORF72*-mutated astrocytes exhibit increased oxidative stress and senescence while secreting paracrine factors to induce oxidative stress in healthy MNs (Birger et al., 2019).

To combine these cell types *in vitro*, a compartmentalized ALS-on-a-chip model was developed to co-culture engineered 3D SkM tissues with heterogeneous (MNs and astrocytes) hiPSC-derived neural spheroids within a microfluidic device (Osaki et al., 2018). Functional NMJs were formed by axonal outgrowth from spheroids into SkM and light was used to stimulate the channelrhodopsin-2–expressing MNs to induce muscle contraction (Osaki et al., 2018). Engineered ALS motor units within this system exhibited higher degradation rates, induced fewer muscle contractions, and increased SkM apoptosis. These features were reversed through treatments with rapamycin and/or bosutinib as potential therapeutic agents (Osaki et al., 2018). A microfluidic compartmentalized 2D co-culture system made of hiPSC-MNs derived from three ALS mutant lines and primary wild-type myotubes exhibited axonal varicosities, limited axonal growth, and increased excitability (Guo et al., 2020b). Functional NMJs with ALS MNs were decreased in number and fidelity and showed increased fatigue index, while the Deanna protocol nutritional supplementation was found to correct these deficits in all lines (Guo et al., 2020b).

To improve upon our understanding of the underlying mechanisms of ALS pathology and to develop curative therapies, it will be critical to complement studies in both animal models and *in vitro* systems. ALS animal models with a wide variety of mutations have allowed for significant advancement in our understanding of the disease. However, limited disease severity and phenotypes, especially within supporting cells such as astrocytes (Oberheim et al., 2009), are likely to undermine clinical success of ALS therapeutics validated in animal models. Increasing disease severity in mice by additional knockdown of SOD1 in astrocytes, assessing the impact of different genetic backgrounds, and increased physical activity via treadmill running or swimming may yield more translationally relevant *in vivo* ALS models. For *in vitro* studies, current ALS-on-a-chip models (Osaki et al., 2018; Guo et al., 2020b) hold great potential for clinically relevant disease modeling and predictive drug screening as they exhibit therapeutically reversible ALS phenotypes and utilize platforms that support high-throughput studies. However, these models have yet to be analyzed to assess if they appropriately recapitulate pathological decrease in synaptophysin concentration, marked denervation, shrunken nerve terminals, and flattened synaptic clefts observed in ALS patients (Bjornskov et al., 1975; Tsujihata et al., 1984; Yoshihara et al., 1998). Further developments to *in vitro* ALS human models will be required to better recapitulate ALS pathology and disease progression. First, modular ALS platforms combined with non-invasive longitudinal and functional assessments can be utilized to help answer the fundamental question if ALS disease progression is due to direct loss of cortical MNs or their retrograde degeneration originating from muscle. Second, improved methodologies to generate hiPSC-derived MN subtypes will allow for further investigations into the preferential subtype-specific MN degradation observed in ALS (Tremblay et al., 2017). Third, incorporation of supportive cell types such as SCs will create a more realistic model of disease as SCs from ALS patients exhibit abnormal morphology with disorganized processes that extend into synaptic clefts (Bruneteau et al., 2015). In *SOD1* mouse studies, similar disorganized processes were seen within SCs alongside upregulated galectin-3, a marker of phagocytosis (Martineau et al., 2020). These extensions may block the synaptic cleft, disrupting MN-SkM communication and contributing to ALS pathology, offering an interesting area for investigations within *in vitro* models.

### Myasthenia Gravis

Myasthenia gravis is a rare autoimmune disorder characterized by accelerated fatigue within voluntary muscles, primarily in extraocular and facial muscles, proximal limbs, and neck extensors (Jayam Trouth et al., 2012). Weakness is highly variable and worsened by a variety of factors including heat, stress, and exercise (Jayam Trouth et al., 2012). MG is associated with accumulation of autoantibodies against NMJ proteins including muscle specific tyrosine kinase (MuSK), low-density lipoprotein receptor-related protein 4 (LRP4), and, most significantly, the nicotinic acetylcholine receptor (nAChR) (Nacu et al., 2015). The accumulation results from T cell-directed attack upon these postsynaptic membrane proteins (Ha and Richman, 2015). MG is often initially identified by ptosis and can be further classified into several subgroups based on autoantibody type and clinical features (Nacu et al., 2015). There are a wide range of treatments approved for MG including immunosuppression, anticholinesterase drugs, immunomodulation, and thymectomy (Farmakidis et al., 2018), but none provide curative outcomes.

Although the primary investigations of MG have focused on NMJ morphology and function, there have been reports of pathophysiological alterations in SkM. Human muscle atrophy was shown to present early in MG progression (Zouvelou et al., 2012) and accelerated atrophy was seen in type II fibers as compared to type I fibers (Wang et al., 2018). Additionally, accumulated anti-nAChR antibodies have been shown to modulate muscular IL-6 production altering mTOR signaling which may be responsible for MG-associated muscle fatigue (Maurer et al., 2015). Muscle biopsies from MG patients showed increased Pax7^+^ satellite cell pool, while isolated MG myoblasts exhibited increased proliferation and differentiation potential (Attia et al., 2017). A similar increase in satellite cell number, proliferation, and differentiation was observed in anti-nAChR mouse models of MG (Attia et al., 2017). Additionally, these mice exhibited delayed SkM maturation following development indicated by lower MyoG expression, reduced fiber size, and increased embryonic myosin heavy chain expression (Attia et al., 2017).

The role of autoantibodies in MG have been confirmed in multiple animal studies. Rabbits treated with anti-nAChR antibodies showed characteristic MG symptoms, confirming the autoimmune nature of MG (Patrick and Lindstrom, 1973). Similarly, administration of autoantibodies against LRP4 and MuSK in mouse models have reproduced MG symptoms indicating important roles of these receptors in disease (Wang et al., 2018). While useful in understanding NMJ dysfunction, acute induction of symptoms through autoantibody administration does not appropriately mimic the chronic progression of MG (Wang et al., 2018). Furthermore, immunized mice inconsistently exhibit clinically observable weakness, rarely present with extraocular muscle fatigue, and fail to recapitulate the dynamic nature of MG disease severity (Wang et al., 2018). Finally, there are additional auto-antibodies implicated in MG against proteins such as titin (Aarli, 2001), agrin (Gasperi et al., 2014), and cortactin (Gallardo et al., 2014) that are not considered in these models.

As MG is an autoimmune disorder, *in vitro* models of MG can be developed by adding patient serum to an existing NMJ platform. The first example of this utilized a 2D co-culture of optogenetically active hESC-derived MNs with primary myotubes to induce a reversible reduction in muscle contraction amplitude by treatment with IgG or active complement protein from MG patients (Steinbeck et al., 2016). In a 3D co-culture model of hiPSC-derived MNs and engineered SkM tissue, localized deposition of complement C3c protein was shown in AChRs of NMJs and treatment with MG IgG decreased number of MN-activated myofibers (Bakooshli et al., 2019). Additionally, automated optogenetic control was incorporated into a 3D MN-SkM culture system and differential responses to sera from donors were measured according to phenotype severity (Vila et al., 2019). Recently, hPSC-derived axial stem cells have been used to generate human neuromuscular organoids containing MNs, SCs, and SkM and study MG (Martins et al., 2020). This model showed reduced NMJ volumes alongside decreased rate and amplitude of contraction in response to MG sera. Although these models have recapitulated some aspects of MG through addition of patient sera, they are limited as they do not utilize MG patient-derived cells.

Overall, understanding the multi-organ nature of MG will require investigations in both animals and *in vitro* human model systems. While animal models are suitable for investigating the involvement of NMJs and immune system, they do not capture MG disease severity, resulting in failure of multiple MG treatments in clinical trials despite prior validation in animals (Mantegazza et al., 2016). Furthermore, direct analyses of heterocellular interactions important for MG pathology in animal models are hindered by the complexity of *in vivo* environments. On the other hand, *in vitro* models exhibit phenotypic and functional responses to patient sera that match disease severity in human donors (Vila et al., 2019) and can enable unique studies to elucidate the roles of cellular crosstalk in disease progression; however, they do not model systemic pathogenesis. Therefore, the next generation of *in vitro* MG models should incorporate immune cells to better recapitulate the autoimmune inflammatory environment. Specifically, incorporation of autoreactive T-cells would allow for investigations of the pathological development of autoantibodies beyond studying the effect of MG serum addition within existing platforms. In animal models of MG, macrophages can act as antigen-presenting cells and help produce self-AChR antibodies (Kinoshita et al., 1988) and as such, their incorporation into human *in vitro* systems (Juhas et al., 2018) would allow systematic studies of their roles in MG autoimmunity. Finally, further studies of SCs within *in vitro* models of MG should be performed to analyze their localization to the presynaptic membranes and potential neuroinflammatory roles (Ydens et al., 2013), including involvement in clinically observed pathological features such as disorganized axonal microorganelles, accumulation of Reich granules, and lipopigments (Kimura and Nezu, 1989).

### Duchenne Muscular Dystrophy

Duchenne muscular dystrophy, a genetic myopathy with the highest prevalence of 7.1 in 100,000 male births (Crisafulli et al., 2020), is a fatal X-linked disorder caused by mutations in the dystrophin gene (Yiu and Kornberg, 2015). Dystrophin is an integral member of the dystrophin glycoprotein complex (DGC) that transmits contractile forces from the sarcomere to the ECM and functions as a molecular shock absorber (Le et al., 2018). In mature myofibers, dystrophin deficiency leads to sarcolemmal instability (Weller et al., 1990), abnormal calcium homeostasis (Tutdibi et al., 1999), and muscle degeneration (Torres and Duchen, 1987). In SCs, dystrophin regulates muscle stem cell commitment via epigenetic modifications (Chang et al., 2018) and regulation of cell polarity (Dumont et al., 2015b; Wang Y.X. et al., 2019). Loss of dystrophin results in increased levels of myogenic progenitors with an impaired ability to commit to differentiation resulting in diminished regenerative ability (Dumont et al., 2015b; Wang Y.X. et al., 2019). Together, this impaired satellite cell function, impeded muscle regeneration, and constant cycles of muscle degeneration result in progressive muscle weakness, loss of ambulation, and ultimately death due to respiratory failure (Yiu and Kornberg, 2015).

In addition to roles within SkM, dystrophin regulates neuronal function and is vital for healthy NMJ maintenance (Tintignac et al., 2015). Dystrophin and other members of the DGC are enriched at post-synaptic folds of the NMJ (Waite et al., 2009) and stabilize nAChRs (Zaccaria et al., 1998). Dystrophic myofibers are associated with increased rates of NMJ branching, fracturing, and transmission failure (Pratt et al., 2013, 2015a), suggesting a role of dystrophin in NMJ remodeling and/or maintenance. The role of dystrophin at the pre-synaptic level of NMJ structure and function is less clear. Increased pre-synaptic nerve terminal branching, axon sprouting, and denervation have been observed in mdx mice (Pratt et al., 2015b; van der Pijl et al., 2016) and humans (Nagao et al., 2003). These structural changes seen in dystrophic NMJs result in functional deficits including altered EMG characteristics and decreased safety factor of neuromuscular transmission in both patients (Priez et al., 1992) and mice (van der Pijl et al., 2016). Clinically, NMJ dysfunction results in increased sensitivity and slowed recovery from neuromuscular blocking drugs such as rocuronium and mivacurium (Ihmsen et al., 2009), contraindicating their use as anesthetics in DMD patients (Breucking et al., 2000). Lastly, dystrophin also regulates neuronal development and function in the brain which most likely contributes to the increased incidence of neurological abnormalities including autism, attention deficit disorder, and learning disabilities in DMD patients (Ricotti et al., 2016).

The majority of our understanding of DMD has been derived from a range of preclinical animal models (Wells, 2018) with the most common model being the *mdx* mouse which has a naturally occurring nonsense point mutation in exon 23 preventing dystrophin protein expression (Manning and O’Malley, 2015). The *mdx* mice exhibit several expected disease features including fibrosis (Gregorevic et al., 2008), respiratory dysfunction (Burns et al., 2018), cardiomyopathy (Mareedu et al., 2021), metabolic dysfunction (Moore et al., 2020), and muscle weakness (Barton et al., 2005). However, the mdx phenotype is milder and has a slower progression compared to clinical symptoms in DMD patients (Dangain and Vrbova, 1984). Disease severity of the *mdx* mouse model can be increased by the knockout of utrophin, which undergoes compensatory upregulation in *mdx* mice to protect against membrane instability (Deconinck et al., 1997). Alternatively, disease severity can be increased by knockout of the telomerase gene (*mdx*/*mTR*) which shortens telomere length and induces a more severe SkM (Sacco et al., 2010) and cardiac (Mourkioti et al., 2013) pathology. Larger preclinical animal models including rats (Larcher et al., 2014), rabbits (Sui et al., 2018), dogs (Nghiem and Kornegay, 2019), and pigs (Selsby et al., 2015) show greater disease severity and lethality than the traditional *mdx* mouse model. However, ethical concerns (Yokota et al., 2012) and secondary complications such as inability to feed (Gaschen et al., 1999) have limited their use to date. From larger animals, the golden retriever muscular dystrophy model (GRMD) (Nghiem and Kornegay, 2019) has been studied the most, but financial, ethical, and animal number concerns limit its use (Wells, 2018). Nevertheless, these preclinical models played important roles in validating current standard of care glucocorticoid therapy (Hudecki et al., 1993) and eteplirsen, a novel antisense oligonucleotide treatment approved by the FDA in 2016 (Khan et al., 2019).

Despite some success, numerous candidate therapeutics identified in animal studies have failed to be effective in humans driving the development of improved model systems (Rybalka et al., 2020). A primary limitation of current animal models is their low genetic and epigenetic diversity. DMD in humans is caused by over 4700 different mutations, and disease severity and response to pharmacological agents is heavily influenced by a range of disease modifiers such as expression levels of latent TGFB binding protein 4 (LTBP4) (Flanigan et al., 2013) and osteopontin-1 (Kyriakides et al., 2011). Disease severity of the *mdx* mouse model has been improved by crossing the *mdx* mutation onto the DBA/2J background, which contains a pro-fibrotic polymorphism in LTBP4 (Heydemann et al., 2009), resulting in greater fibrosis and functional impairment (van Putten et al., 2019). In the past decade, the development of genome editing tools such as TALEN and CRISPR-Cas9 has enabled the generation of humanized *mdx* mouse models (Aartsma-Rus and van Putten, 2019). These mice specifically model human dystrophin mutations to enable preclinical validation of gene editing therapies using CRISPR-Cas9 (Min et al., 2019) or antisense oligonucleotide (Veltrop et al., 2018) technologies.

While animal models can be genetically modified to include aspects of patients’ genetic diversity, true personalized disease platforms require use of patients’ tissues or cells. *In vitro* 2D studies of mouse or human DMD myoblasts have been utilized to model satellite cell dysfunction (Wang Y.X. et al., 2019), impaired myotube formation (Blau et al., 1983), and altered Ca^2+^ handling (Imbert et al., 1995). Myotubes lacking dystrophin or other members of the DGC display decreased AChR clustering (Kong and Anderson, 1999), indicating that post-synaptic NMJ abnormalities can occur in the absence of neural cells. When engineered into 3D tissues, DMD primary and immortalized patient cells display decreased fusion and force of contraction, atrophic myotubes, and decreased nuclear anisotropy (Nesmith et al., 2016; Al Tanoury et al., 2021; Ebrahimi et al., 2021). Large scale personalized platforms amenable to pharmacological screens will require the use of patient hiPSCs due to ethical and proliferative limitations of muscle biopsy-derived primary cells and a need for non-muscle cells such as MNs (Wang J. et al., 2019). Encouragingly, two chemicals (ginsenoside and fenofibrate) identified to improve fusion rate in hiPSC-derived DMD myoblasts were both found to improve muscle structure and function in *mdx* mice (Sun et al., 2020). Similarly, prednisolone, the current standard of care for DMD patients, rescued fusion, force of contraction, and branching defects in hiPSC-derived DMD myotubes (Al Tanoury et al., 2021), further implying a potentially predictive nature of these *in vitro* assays. In addition to drug development, hiPSC-derived DMD cells from patients with a wide-range of mutations can be utilized for optimization and validation of gene therapies such as guide RNA design for CRISPR-Cas9 mediated genome editing (Min et al., 2019).

Furthermore, hiPSC-based disease models allow building complex tissues to enable studies of the multi-cellular crosstalk in DMD pathogenesis. For example, astrocytes (Patel et al., 2019) and glutamatergic sensory neurons (Ruggieri et al., 2019) generated from DMD hiPSCs displayed increased Ca^2+^ overload and cytoskeletal disorganization indicating that neuronal involvement can be also studied using DMD hiPSC derivatives. Multicellular 3D cultures comprised of hiPSC-derived myoblasts, neurons, endothelial cells, and fibroblasts were successfully generated from both healthy and DMD cells (Maffioletti et al., 2018; Mazaleyrat et al., 2020), but the roles of cellular crosstalk in NMJ function and dysfunction in these cultures remain to be studied. Interestingly, in a primary and immortalized cell line based human 3D co-culture system, endothelial cells were required for DMD fibroblasts to undergo fibrinogenesis, suggesting that complex multicellular 3D platforms may be required to study mechanisms of DMD with high fidelity (Bersini et al., 2018). Recently, a compartmentalized optogenetic neuromuscular DMD model was developed by culturing hiPSC-derived DMD and isogenic control myoblasts with MN spheroids derived from wild-type channelrhodopsin-expressing murine or human ESCs (Paredes-Redondo et al., 2021). In this model, pharmacological inhibition of TGFβ signaling induced partial restoration of AChR and NMJ volumes along with significant up-regulation of MuSK expression (Paredes-Redondo et al., 2021). Besides SkM DMD platforms, hiPSC-derived DMD cardiomyocytes exhibit impaired contractile function (Chemello et al., 2021), altered calcium-handling (Chemello et al., 2021), and mitochondrial dysfunction (Sun et al., 2020), and have been utilized for *in vitro* studies of drug (Lin et al., 2015) and gene (Kyrychenko et al., 2017; Long et al., 2018; Chemello et al., 2021) therapies for DMD.

Since DMD arises from a range of mutations and its progression strongly depends on a variety of disease modifiers, complementary studies in animal and *in vitro* models will be required for improved understanding of disease mechanisms and development of effective therapeutic approaches. While translational relevance of current animal models is limited by low genetic and epigenetic diversity, genome editing technologies such as CRISPR can now enable improved phenotypic and mutational representation in animals (Pickar-Oliver et al., 2021). Regardless of these improvements, use of patient-derived cells will be necessary for truly personalized disease modeling. Recent 3D NMJ DMD models have provided a useful platform for studying NMJ dysfunction *in vitro* (Paredes-Redondo et al., 2021), but fully patient-specific DMD NMJ models remain to be developed. In these models, it will be important to perform careful morphological analysis of axonal branching and NMJ fragmentation to evaluate how well patient phenotypes are recapitulated (Pratt et al., 2013, 2015a). Similar to ALS models, the DMD platforms can be utilized in modular fashion to determine roles of distinct cellular and environmental components in NMJ pathology. For example, SCs within the *mdx* model exhibit disorganized processes directed away from endplates which may block innervation, indicating a potential role of SCs in DMD pathology (Personius and Sawyer, 2005) which remains to be studied *in vitro*. DMD patient and mouse muscles are also characterized by pro-inflammatory immune cell infiltration, which results in increased levels of neutrophils, T cells, and macrophages. Replicating this pro-inflammatory milieu *in vitro* could shed novel mechanistic insights into the effects of inflammation on NMJ structure and function in DMD. While the altered inflammatory milieu is thought to stimulate FAP proliferation and fibrosis (Juban et al., 2018), current DMD models do not exhibit fibrotic changes, thus the incorporation of both inflammatory and FAP cells will likely be essential to fully replicate the advanced stages of disease.

### Myotonic Dystrophy

Myotonic dystrophy is the most prevalent form of muscular dystrophy in adults and is classified into Type I (DM1) and II (DM2). DM1 results from a series of CTG repeats in the DM protein kinase (DMPK) gene, while DM2 results from a series of CCTG repeats in the Zinc Finger 9 (ZNF9) gene (Thornton, 2014). The major pathogenic consequence of these DNA tandem repeats is gain-of-function of the resulting mutant RNA that form hairpin-like structures which bind and sequester RNA-binding proteins (Brouwer et al., 2009). This results in the sequestering and dysregulation of splice factors such as musclebind-like (MBNL) and CUG-binding proteins (CUGBP) (Fernandez-Costa et al., 2011). Ultimately, this leads to alternative splicing of multiple mRNAs including members of the DGC (Nakamori et al., 2007) and t-tubule proteins (Fugier et al., 2011) which contributes to the characteristic progressive myopathy and myotonia in DM. DM1 and 2 have a greater impact on fast muscle fibers and are associated with variable muscle fiber diameter, fiber splitting, and fibrofatty replacement (Vihola et al., 2003). Histologically, DM2 can be distinguished by pyknotic nuclear clumps that occur before the onset of muscle weakness (Meola and Cardani, 2015).

Aberrant splicing is not limited to SkM but occurs in multiple organs including the cardiac and neurological systems (Lee and Cooper, 2009). In DM1, splicing factors in the MBNL family accumulate within ribonuclear foci within both pre-synaptic MNs and post-synaptic nuclei leading to NMJ instability (Wheeler et al., 2007). DM1 patients exhibit thinned axon and myelin sheets without denervation (Fardeau and Tome, 1980). Additionally, repetitive nerve stimulation and single fiber electromyography in DM1 patients show abnormal nerve conduction and pathological jitter suggesting NMJ instability (Bombelli et al., 2016). DM1 mouse models exhibiting pathophysiological levels of CTG repeats in the DM1 region show distal denervation of diaphragm NMJs, reduced AChRs on the post synaptic membrane, and loss of unmyelinated fibers (Panaite et al., 2008). Furthermore, variable levels of axonal neuropathy (17–46%), axonal loss, and myelin sheath thinning have been reported in both DM1 patients (Peric et al., 2013) and mice (Panaite et al., 2011). Functionally, these structural alterations lead to abnormal nerve conduction and pathological jitter in DM1 patients (Bombelli et al., 2016). Currently there are no curative therapeutics for either DM1 or DM2 (Pascual-Gilabert et al., 2021). However, nearly two dozen preclinical and clinical drug development programs are currently active encompassing repurposed drugs, gene therapy, oligonucleotide therapeutics, and novel chemical treatments (Pascual-Gilabert et al., 2021).

Several animal models have been developed to study DM1 disease mechanisms and investigate potential therapies. DM1 was first modeled in DMPK knockout mice which only developed mild myopathy (Jansen et al., 1996), mild cardiac conduction dysfunction (Berul et al., 1999), and failed to replicate the multisystemic patient phenotype (Jansen et al., 1996). Through the overexpression of DMPK with toxic CTG repeats, a stronger disease phenotype was developed with ribonuclear foci changes, SkM atrophy, slowed growth, weakness, and myotonia (Vignaud et al., 2010). Neurologically, these mice exhibit RNA toxicity within Bergmann glia and Purkinje cell hyperexcitability, and reduced motor coordination representative of DM1 patients (Sicot et al., 2017). However, they still exhibit mild splicing defects and disease phenotype compared to patients (Huguet et al., 2012). Alternatively, myotonia and alternative splicing defects can be induced by combined MBNL1 inactivation and expression of untranslated CUG (HSA_*LR*_ model), but this does not lead to muscle wasting or denervation (Wheeler et al., 2007). Overexpression of CUGBP1 causes more severe myopathy and cardiomyopathy but is limited by high mortality and breeding issues. Through no individual mouse models capture all disease features or fully recapitulate severity seen in patients, they have provided significant mechanistic insights into the genetic causes of specific disease phenotypes.

Due to the multisystemic nature of DM, multiple muscle and non-muscle cell lines have been utilized to study DM pathology (Matloka et al., 2018). HEK, HeLa, and C2 cells with CTG repeats inserted in the 3′UTR of a truncated *DMPK* gene have replicated splicing misregulations and ribonuclear foci phenotypes (Philips et al., 1998; Warf and Berglund, 2007). *In vitro* drug screens to ameliorate these phenotypes have been utilized to identify novel therapeutics for DM (Warf and Berglund, 2007; Konieczny et al., 2017). DM patient-derived primary myoblast cultures exhibit metabolic alterations (Renna et al., 2017), splicing abnormalities (Laustriat et al., 2015), and ribonucleic foci formation (Fardaei et al., 2002). Myotube cultures have also been generated from MyoD overexpression in DM fibroblasts (Kuyumcu-Martinez and Cooper, 2006; Ravel-Chapuis et al., 2012) and hiPSCs (Gao et al., 2016; Ueki et al., 2017; Martineau et al., 2018) from DM patients. Similar to primary myoblasts, these cells exhibited RNA splicing abnormalities and formation of ribonucleic foci. Recently, the first 3D *in vitro* human muscle model of DM1 was developed by encapsulating patient-derived fibroblasts overexpressing MyoD in micromolded gelatin methacryloyl-carboxymethyl cellulose methacrylate hydrogels (Fernández-Garibay et al., 2021). Furthermore, for studies of neuromuscular abnormalities, DM patient ESCs were differentiated into MNs and co-cultured together with healthy SkM (Marteyn et al., 2011) and found to exhibit diminished synaptogenesis and increased neurite outgrowth associated with low expression of genes in the *SLITRK* family.

Additionally, hiPSC lines derived from DM patients have been used to study pathological alterations in distinct cell types (Gao et al., 2016; Ueki et al., 2017; Martineau et al., 2018). The neurological component of DM1 has been studied in hiPSC-derived neurons and astroglia which display expected ribonucleic foci and splicing abnormalities. These models have been used to demonstrate proof-of-principle phenotypic reversal through genome editing (Xia et al., 2015). While all models demonstrate histological alterations, no *in vitro* studies have shown myogenic or neuronal functional deficits. Additionally, CTG repeats have been shown to be unstable in pluripotent cells (Du et al., 2013) and CTG repeats do not expand when naive hiPSCs are differentiated into cardiomyocytes, muscle, or neurons as seen *in vivo*. Recently, DM1 hiPSC-derived cardiomyocytes from DM1 patients with varied CTG repeat lengths exhibited toxic RNA foci and mis-spliced *MBNL1/2* transcripts and showed two distinct ions channel (Na^+^ and Ca^2+^) perturbations (Poulin et al., 2021). This platform revealed the underlying mechanism of electrical cardiac alterations in DM1 and can be used in the future to validate potential therapeutics in a high throughput fashion by monitoring action potential propagation and ionic currents in the human DM1 cardiomyocytes.

Overall, translationally relevant DM modeling is complicated by the multifaceted influences of DM throughout the entire body, most notably within neurological and muscular tissues. Although mouse models have greatly expanded our understanding of this pathogenic RNA disease, no single mouse model has exhibited severity comparable to patients or encompassed the myriad of DM phenotypes present *in vivo*. For example, DM1 patients exhibit toxic RNA accumulation whereas HSA_*LR*_ mice do not, which may contribute to the lack of denervation-like features in these mice (Wheeler et al., 2007). Intercrossing mouse lines may improve recapitulation of DM pathology and even show pathological synergy between symptoms. However, further development of patient-derived *in vitro* NMJ models will be critical for the ability to directly analyze the human DM NMJ. First, further optimization of hiPSC culture and differentiation protocols to produce myogenic and neuronal cells with stable CTG repeats that exhibit functional deficits will be critical. Second, novel methods for differentiating specific muscle subtypes from hiPSCs will augment our ability to accurately model and study DM as it preferentially affects fast muscle fibers (Vihola et al., 2003). Third, in DM1 patients, SCs exhibit abnormal glycogen accumulation and crystalline structures within their processes (Borenstein et al., 1977) and may contribute to disease progression through unknown mechanisms which remain to be studied in NMJ-SC co-cultures. Fourth, DM1 patients and mice show increased pro-inflammatory gene signatures and upregulation of the IL-6 pathway (Nakamori et al., 2017) and tumor necrosis factor superfamily member 12 (TNFSF12) signaling (Yadava et al., 2015). Incorporation of immune cells in DM NMJ models would allow important studies of how inflammation may contribute DM progression.

### Pompe Disease

Pompe disease, also known as glycogen storage disease type II (GSDII), is a rare metabolic autosomal recessive disorder that results from deficiency of acid α-glucosidase (GAA) (Reuser et al., 1995). Pompe disease is categorized into two major types based upon disease onset and GAA enzyme activity, although a continuous spectrum of phenotypes exists. Infantile-onset Pompe disease (IOPD) is caused by very low GAA enzyme activity and results in fatal cardiac, neurologic, hepatic, and muscular dysfunction between ages 1 and 2 (Lim et al., 2014). In contrast, late-onset Pompe disease (LOPD) involves higher GAA activity which results in slower disease progression (Chan et al., 2017). GAA breaks down lysosomal glycogen, and its dysfunction leads to intralysosomal glycogen accumulation in various tissues but most abundantly within skeletal and cardiac muscle (Reuser et al., 1995). The build-up of glycogen in striated muscle leads to lysosomal enlargement, vacuolation (Prater et al., 2013), autophagosome proliferation (Nascimbeni et al., 2015), and lipofuscin aggregation (Feeney et al., 2014). These alterations are hypothesized to disrupt cellular and sarcomere architecture resulting in progressive muscle weakening and ultimately respiratory or cardiac failure (Reuser et al., 1995). However, neuromuscular abnormalities such as increased neuromuscular jitter and variance in response latency are seen within Pompe patient muscle prior to muscle weakness (Stålberg and Trontelj, 1997). This suggests that neurological dysfunction could be a key driver of muscle weakness and myopathy in Pompe disease.

In Pompe patients and mice, glycogen accumulation occurs in the brain (Mancall et al., 1965; Lee et al., 2011), central nervous system (CNS) (Mancall et al., 1965; Martini et al., 2001; DeRuisseau et al., 2009), and MNs (Gambetti et al., 1971; DeRuisseau et al., 2009). Furthermore, Pompe MNs have three-fold higher soma size (Mancall et al., 1965) and are more apoptotic (Turner et al., 2016), resulting in decreased motor output and neuronal loss (DeRuisseau et al., 2009). Pompe mice also exhibit significant NMJ alterations due to both presynaptic changes, such as reduced myelin thickness and neurofilament proteins, and postsynaptic changes, such as NMJ fragmentation (Falk et al., 2015). These structural alterations result in impaired neural output including increased numbers of denervated NMJs (Falk et al., 2015), decreased burst amplitude (DeRuisseau et al., 2009), and increased spontaneous EMG activity (Hobson-Webb et al., 2011). In addition to MNs, structural alterations and glycogen accumulation are found in neuronal support cells including astroglia and SCs (Martin et al., 1973). These alterations can lead to secondary disease traits including cognitive declines (Spiridigliozzi et al., 2017), speech disorders (Muller et al., 2009), and sensorineural and/or conductive hearing loss (Prater et al., 2012) demonstrating the wide ranging impacts of GAA deficiency.

Pompe disease has been predominantly modeled in the GAA knockout mice (Raben et al., 2001) that recapitulate key disease features such as striated muscle and nervous tissue glycogen accumulation (DeRuisseau et al., 2009), lysosomal abnormalities (Doyle et al., 2019), neuropathology (DeRuisseau et al., 2009), cardiac defects (Han et al., 2016), and muscle weakness (Lee et al., 2018). However, despite a complete lack of GAA, these mice exhibit a LOPD phenotype with late onset, slow disease progression, and normal breathing in normoxic conditions (Han et al., 2016; Gatto et al., 2017). Recently, crossing the GAA KO mice to the DBA2/J background resulted in a much more severe disease phenotype including early lethality, respiratory defects during normoxia, and more severe cardiomyopathy (Colella et al., 2020). While the pro-fibrotic polymorphism in LTBP4 found in the DBA2/J background has not been implicated in Pompe disease, polymorphisms in angiotensin-converting enzyme (ACE) and alpha-actinin 3 (ACTN3) (De Filippi et al., 2014) impact disease onset within LOPD. The classical GAA KO mouse model has been essential for identifying and validating potential therapeutics (Doyle et al., 2019) and has allowed for deeper understanding of the roles of the mTOR pathway, lysosomal dysregulation, and autophagocytosis in Pompe disease progression (Han et al., 2016; Gatto et al., 2017). In addition to mouse models, a novel zebrafish model, in which GAA activity is significantly reduced but not totally absent, displays significant motor behavior and NMJ abnormalities (Bragato et al., 2020). When this model was used as a drug screening platform, 3-bromopyruvic acid (Bragato et al., 2020) and 3,4-diaminopyridine phosphate (Cinzia et al., 2021) were found to increase AChR abundance, improve NMJ structure, and recover typical movement patterns. A baboon model of Pompe disease is currently used as a large animal preclinical model which has shown utility for therapeutic evaluation (Rastall et al., 2016). However, considering nearly 600 reported mutations within Pompe patients and the impact of disease gene modifiers (De Filippi et al., 2014), these models are unable to fully recapitulate human genetic complexity of the disease (Fukuda et al., 2007).

Current clinical therapy for Pompe disease is enzyme replacement therapy (ERT) which systemically delivers the recombinant human GAA (rhGAA) to break-down accumulated glycogen. While ERT significantly prolongs patient lifespan and augments quality of life (Kishnani et al., 2009), it is limited by inefficient delivery to SkM tissues (van der Ploeg et al., 2010), neutralization by host antibodies (De Vries et al., 2017), high dose requirements (Chien and Hwu, 2007), variable patient response (Kishnani et al., 2010), and high cost ($300,000 per year) (Güngör et al., 2013). Importantly, rhGAA does not appear to impact disease phenotypes in neurons nor does it cross the blood-brain barrier to help treat other neurological disease symptoms. Furthermore, restoration of SkM GAA activity does not restore nerve-invoked contractile function in GAA KO mice suggesting therapies should target neuronal tissues (Falk et al., 2015). In support, neuron-specific gene therapy in Pompe mice improved motor coordination, decreased astrogliosis, and increased myelination (Lee et al., 2018). However, early administration of AAV9-hGAA (age 1 month) led to the greatest restoration of GAA activity and overall function, while late administration (age 15 months) was not effective, reflecting how this treatment was unable to reverse a deficit in NMJ function and force production despite removing muscular glycogen accumulation (Todd et al., 2015).

In addition to studies in animal models and patients, *in vitro* human cell culture systems play important roles in modeling genetic diversity and neuromuscular dysfunction in Pompe disease. Specifically, human myotube cultures accurately model clinical differences between IOPD and LOPD patients (Raben et al., 2010), with IOPD myotubes displaying lysosomal enlargement (Spampanato et al., 2013) and LOPD myotubes exhibiting autophagosome accumulation (Nascimbeni et al., 2012). Glycogen accumulation and lysosomal enlargement in primary and hiPSC-derived IOPD myotubes could be prevented by overexpression of transcription factor EB (TFEB), a regulator of autophagy and lysosomal biogenesis (Spampanato et al., 2013; Sato et al., 2016). These findings were translated to GAA KO mice, where AAV delivery of TFEB ameliorated muscle pathology and restored contractile function (Gatto et al., 2017). Recently, the first *in vitro* 3D model of human Pompe disease SkM was reported using primary muscle cells (Wang et al., 2021). This model exhibited the expected lack of GAA activity, increased glycogen levels, lysosomal enlargement, and transcriptomic changes characteristic of Pompe disease but displayed no innate functional weakness (Wang et al., 2021). However, functional deficits could be induced by causing lysosomal stress with chloroquine, enforcing glycogen utilization by glucose starvation, or glycogen phosphorylase inhibition (Wang et al., 2021). Compared to 2D models, this biomimetic platform better recapitulates the *in vivo* Pompe phenotype for studies of glycogen accumulation and responses to candidate pharmacological and gene therapies.

Like other myopathies, large-scale and patient-specific studies of Pompe disease will require the utilization of hiPSCs. Encouragingly, hiPSC-derived myotubes generated through MyoD overexpression (Sato et al., 2016) or directed differentiation exhibit expected reductions in GAA activity and glycogen accumulation (van der Wal et al., 2017). Their utility as drug screening platforms has been shown by the ability of recombinant GAA (Yoshida et al., 2017), lentivirus encoding GAA (Sato et al., 2016), and antisense oligonucleotide (van der Wal et al., 2017) treatments to recover GAA enzyme activity and normalize cellular glycogen levels. The CNS involvement in Pompe disease has been modeled *in vitro* with hiPSC-derived neural stem cells which exhibited glycogen and secondary lipid accumulation, increased apoptosis, and enlarged lysosomes (Huang et al., 2019; Cheng et al., 2021). Lysosome size and glycogen accumulation could be decreased with rhGAA, hydroxypropyl-β-cyclodextrin, antioxidants (δ-tocopherol and ebselen), or PI3-K inhibitors (wortmannin and PX-866) (Huang et al., 2019; Cheng et al., 2021). Importantly, ebselen was able to increase GAA activity in the brain of GAA KO mice suggesting that these *in vitro* platforms could identify compounds that target neuronal tissues in Pompe patients (Huang et al., 2019). However, modeling of Pompe NMJ structure and function in hiPSC platforms has not been reported to date.

Overall, Pompe disease causes systemic accumulation of glycogen leading to a myriad of symptoms including cognitive decline, muscular wasting, and NMJ dysfunction. While animal models have been critical for therapeutic developments and understanding Pompe pathology, they do not recreate human genetic complexity. Further complications arise from variations in clinical features between IOPD and LOPD patients underlining the importance of complementing animal studies with *in vitro* disease modeling platforms. Promisingly, hiPSC-derived Pompe disease myotubes exhibit expected reductions in GAA activity and glycogen accumulation and can be utilized for personalized drug screening (Sato et al., 2016; van der Wal et al., 2017; Yoshida et al., 2017). However, current *in vitro* models of 3D Pompe muscle do not model functional weakness in the absence of exogenous stressors such as those inducing lysosomal deficiency or glucose starvation (Wang et al., 2021). Functional weakness could potentially be gained by increasing experimental duration or muscle maturation as done in 2D micropatterned cultures that increased pathological LAMP1-positive lysosome accumulation in hiPSC-derived Pompe myotubes (Jiwlawat et al., 2019). Alternatively, the lack of baseline functional deficit in engineered Pompe muscles could point to the requisite NMJ involvement, warranting the development of hiPSC-derived NMJ models of Pompe disease. Additional incorporation of SCs and microglia would be of particular interest as these cells also show glycogen accumulation and cytoplasmic ballooning (Martin et al., 1973) and can contribute to Pompe pathogenesis. Since reduced myelin is seen in Pompe mice (Falk et al., 2015), effects of SC myelination should be also assessed in human *in vitro* models. Finally, ERT is more effective in fast than slow muscle fibers (Hawes et al., 2007), thus the ability to differentiate specific muscle fiber subtypes from Pompe hiPSCs would further augment translational relevance of these *in vitro* models.

## Discussion

Decades of work using both *in vitro* and *in vivo* models of NMDs have led to important mechanistic insights and notable therapeutic advances. Here, we have compared the utility of these models for studying the NMJ structure and function and discussed the current state of disease modeling in the context of five specific NMDs. Animal models have been essential for our understanding of the clinical features of NMDs, but their limited genetic diversity and non-human physiology hinder their ability to fully recapitulate human NMD phenotypes, severity, and progression. Advances in genome editing technologies have facilitated generation of animal models with human mutations, providing a means to generate improved preclinical models for testing pharmacological and gene therapies for NMDs. Nevertheless, truly personalized disease modeling that accurately represents patient genetic and epigenetic diversity will require development of high-fidelity *in vitro* human NMJ platforms.

**FIGURE 6:**
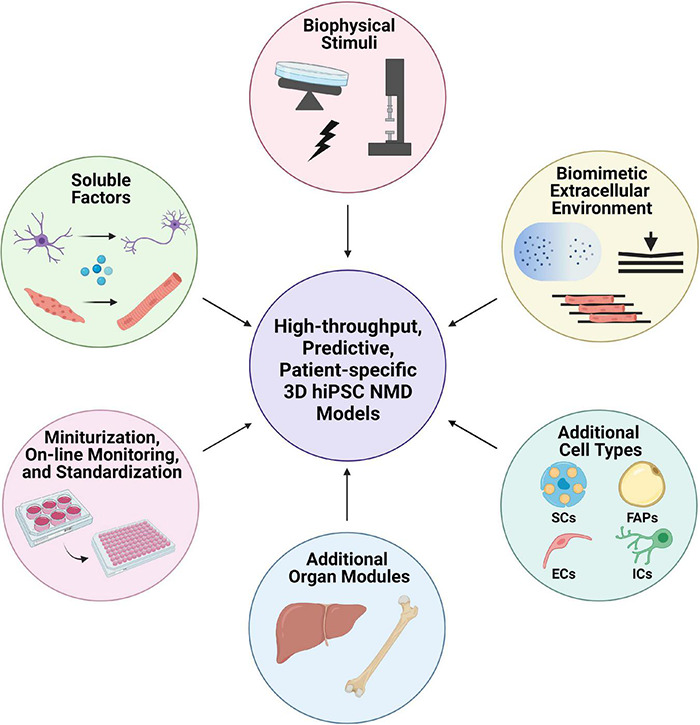
Future developments of hiPSC-derived NMD models. Optimization of soluble factor treatments will be needed to improve maturity of MNs, SkM, and NMJs toward their respective *in vivo*-mimetic phenotypes. Incorporation of biophysical stimulation during cell culture such as electrical stimulation, mechanical stretch, and media agitation are expected to further improve functional maturity of NMJs. Biomaterial design and microfabrication/microfluidics techniques can be applied to generate biomimetic microenvironments for cell growth via controlled presentation of developmentally relevant gradients in growth factors, topography, and stiffness. As the function of native NMJs relies on complex multi-cellular interactions, the ability to incorporate Schwann cells (SCs), endothelial cells (ECs), fibro-adipogenic progenitors (FAPs), and immune system cells (ICs) would improve modeling of *in vivo* states and our understanding of NMJ physiology and pathology. Incorporation of additional organ compartments sharing culture media with NMJs, such as intestine, gut, and liver will better emulate human drug responses, while adding bone, ligament, cartilage, and fat compartments will allow studies of the organ-organ crosstalk in NMDs. Finally, further miniaturization and standardization of culture systems along with development of non-invasive metabolic and functional readouts will enable establishment of versatile NMD platforms for high-throughput drug discovery research.

Recent advances in tissue-engineering methodologies have increased our *in vitro* modeling capabilities and furthered our understanding of human NMDs. hiPSC-derived models, in particular, hold promise for use in large-scale pharmaceutical testing, systematic analysis of disease mechanisms, and development of patient-specific treatments. However, additional progress will be needed to fully recapitulate NMD progression and complexity *in vitro* to allow for meaningful studies of underlying pathological mechanisms and drug responses (Figure 6). Specifically, hiPSC-derived NMJ models remain immature compared to primary NMJ models even after lengthy differentiation protocols. Therefore, improved differentiation methods will be necessary to not only replicate but accelerate developmental processes to obtain hiPSC-derived MNs and SkM cells and generate NMJs with mature structure and functionality akin to those of native motor units. Modifications of existing differentiation protocols to derive specific MN subtypes and SkM fiber types and stimulate NMJ maturation (Zhang et al., 2016) will allow development of *in vitro* models that can investigate why specific types of NMJs are distinctly affected by different NMDs and will enable targeted pharmacological testing of most affected tissues, such as type II muscle fibers in MG (Wang et al., 2018).

Replicating native, mature functional properties of NMJs will be crucial for accurate *in vitro* modeling of NMDs that often occur in adulthood. The immature state of hiPSC-derived cells remains a significant hurdle to generating predictive *in vitro* disease models or developing safe and effective regenerative therapies. Development of high-fidelity human NMJ models of NMDs is further complicated by the lack of detailed histological and functional descriptions of NMJs in NMD patients. Therefore, evaluation of *in vitro* human NMJ function and dysfunction as well as early formation relies largely on comparisons with murine models of development and disease. On the other hand, duration of human MN neurogenesis and maturation *in vivo* (Gogliotti et al., 2012; Stein et al., 2014) and *in vitro* (Johnson et al., 2007; Wainger et al., 2014) is substantially longer compared to murine counterparts, requiring longer-lasting and costly *in vitro* protocols to achieve mature MN states. Accelerating human MN maturation is commonly performed via small molecule inhibition of Notch. Disruption of Notch signaling accelerates neuronal differentiation by delaying the cell cycle transition from G1 to S phase increasing the commitment of progenitors toward neurogenesis (Crawford and Roelink, 2007; Borghese et al., 2010) and pre-MNs toward a post-mitotic, mature MN fate (Maury et al., 2015). While the small molecules that stimulate cell cycle exit have proven successful in accelerating MN maturation, their effects on the acquisition of distinct MN fates (e.g., MMC, SAC, PMC, LMC, HMC, PGC, ANS) are unexplored. As different MN subtypes emerge at distinct developmental stages *in vivo*, premature cell cycle exit could obstruct the ability to activate later MN subtype programs, which will require future investigations.

Similar to MNs, the immature state of current engineered SkM tissues lessens their physiological relevance and utility in disease modeling. A number of methods have been employed to enhance *in vitro* SkM maturation including dynamic culture (Juhas and Bursac, 2014), mechanical stretch (Powell et al., 2002), electrical stimulation (Khodabukus et al., 2019), growth factors (Ebrahimi et al., 2018), hormones (Butler-Browne et al., 1984), and small molecules (Selvaraj et al., 2019). Interestingly, some of these same interventions are also beneficial to both MN and NMJ formation, maturation, and regeneration. For example, electrical stimulation increases axon regeneration and recovery of motor function in sciatic nerve injury in Sprague-Dawley rats (Fu et al., 2020) and improves alignment and maturation of human myotubes in culture (Ahadian et al., 2012; Khodabukus et al., 2019). As functional innervation is required for muscle maturation and mature muscle contributes to maintaining functional NMJs, it will be important to develop methods that will improve overall maturation state in NMJ co-cultures. Additionally, myonuclei are typically localized at the periphery of muscle fibers, but specialized myonuclei are anchored below the mature postsynaptic membrane *in vivo* (Grady et al., 2005). Advances in single-nucleus RNA sequencing have revealed hundreds of novel genes expressed within postsynaptic myonuclei (Petrany et al., 2020). Genetic knockdown of a small subset of these genes revealed Gramd1b as a positive and Ufsp1 as a negative modulator of AchR clustering suggesting that novel insights into NMJ maturation and maintenance can be obtained from sequencing datasets. Similar studies of transcriptional specialization within modular neuromuscular cultures (consisting of multiple compartments and cell types) would improve our understanding of NMJ development leading to effective methods to increase NMJ maturation.

Ultimately, besides accelerated maturation of MNs and SkM cells, the incorporation of additional non-myogenic muscle-resident cell types, such as SCs, fibro-adipogenic progenitors (FAPs), and endothelial cells will be required to recreate native NMJ complexity. Here, heterocellular interactions can stimulate formation and advanced maturation of biomimetic NMJs to model both normal physiology and disease. For example, SCs are essential in the formation and maintenance of the adult NMJ (Ko and Robitaille, 2015) and their incorporation into NMJ models has supported MN and SkM viability, myelinated axons, and extended culture times (Singh and Vazquez, 2019; Hyung et al., 2021). Unfortunately, they have been underrepresented within NMD models despite their suggested disease-modifying roles in ALS (Bruneteau et al., 2015), MG (Kimura and Nezu, 1989), DMD (Personius and Sawyer, 2005), DM (Borenstein et al., 1977), and Pompe disease (Martin et al., 1973). Similarly, FAPs reside adjacent to NMJs, neighboring SCs and MNs, where they actively influence SCs and maintain NMJ integrity (Hogarth et al., 2019; Uezumi et al., 2021). Subpopulations of FAPs are dysregulated in many NMDs and accumulate within atrophied ALS mouse muscles (Gonzalez et al., 2017), exhibit varied gene signatures in response to denervation and cardiotoxin injury (Madaro et al., 2018), and activate IL-6-STAT3 signaling in response to denervation that may lead to fibrosis related to NMDs (Madaro et al., 2018). Vascular cells have critical roles in NMJ development, maintenance, and regeneration *in vivo* (Sawada et al., 2014) as well as secrete neurotrophic factors that support axonal growth *in vitro* (Grasman and Kaplan, 2017). Their roles in NMJ formation and function also remain to be explored and further elucidate their potential roles in NMDs.

Generation of advanced *in vitro* NMD models and platforms for predictive drug screening will also require the incorporation of cell of the immune system and cells involved in drug metabolism, respectively. The immune system is a critical regulator of both neurological (Prinz and Priller, 2017) and muscular (Farup et al., 2015) homeostasis, injury response, and pathology. The chronic injury cycles characteristic of multiple NMDs result in persistent and increased infiltration of monocytes/macrophages (Acharyya et al., 2007), T lymphocytes (Uzawa et al., 2021), and mast cells (Trias et al., 2017) at NMJs. These cells secrete multiple factors that induce a chronic pro-inflammatory environment and contribute to disease progression through several mechanisms that increase oxidative stress and alter stem cell function. Similarly, accurate modeling of autoimmune diseases such as MG will require incorporation of T and B lymphocytes to model chronic inflammation and autoantibody production (Uzawa et al., 2021). Realistic *in vitro* modeling of pharmacodynamics and expected drug concentrations in the bloodstream will necessitate incorporation of additional organ-on-chip (OOC) modules containing extramuscular tissues such as intestine, gut, liver, and fat. These multiplexed culture systems will be able to identify unexpected drug toxicities due to organ-organ crosstalk, such as that seen for bleomycin cardiomyocyte toxicity only in the presence of lung tissue (Skardal et al., 2017). Multiplexed OOC platforms will also enable studies of the more intimate tissue-tissue relationships between skeletal muscle and bone, ligament, or cartilage, which can be perturbed in NMDs as well as in various musculoskeletal degenerative diseases. In support of the feasibility of these complex systems, up to 10 unique OOC modules have been successfully interconnected to form a body-on-a-chip (BOC) platform (Novak et al., 2020). While additional tissue maturation within each OOC module is required, BOC platforms would have the unique potential to permit modular crosstalk studies allowing understanding of the NMD progression on a systemic level.

Beyond incorporation of additional cell types, further advancements in biomaterial design and microfabrication technologies will be critical for successful engineering of microphysiological systems that replicate *in vivo* 3D microenvironments of developing or diseased NMJs. For example, 3D hydrogels that incorporate the basement membrane proteins laminin and collagen IV have been shown to support increased contractile force generation in muscle-only (Hinds et al., 2011) and muscle-neuron (Vilmont et al., 2016) culture platforms and uniquely enable tau aggregation in organoid models of Alzheimer’s disease (Choi et al., 2014). Recent advancements in the design of smart biomaterials that allow spatiotemporal control of growth factor, topographical, and stiffness gradients (Darnell and Mooney, 2017; Kowalski et al., 2018), combined with use of microfluidic devices to establish tissue compartmentalization and gradients of soluble factors (Sun et al., 2019), offer opportunities to precisely influence the migration, proliferation, differentiation, and maturation of hiPSC derivatives through mimicry of the native ECM architecture and biophysical and biochemical cues. For example, 3D bioprinting (Kang et al., 2016; Zhang et al., 2018) of bioactive biomaterials to enable compartmentalized growth and differentiation of MNs and SkM (Osaki et al., 2018; Narayanan et al., 2021), along with microfluidic approaches to establish a local microenvironment supportive of NMJ formation and maturation via spatially defined delivery of auxiliary cells and agrin (Tourovskaia et al., 2008), could provide means to generate highly functional NMJs amenable to rigorous studies of NMD pathology.

Current *in vitro* NMJ models are mainly utilized for small-scale, stand-alone studies, or to supplement whole-organ and organism-level *in vivo* investigations with human cell- and tissue-specific data. Combining animal studies from a single genetic background with *in vitro* validations in hiPSC-based models from genetically diverse patients are likely to improve the predictive value of preclinical therapeutic tests. Eventually, however, advances in hiPSC technology along with miniaturization and standardization of microphysiological systems are expected to enable self-sufficient, high-throughput *in vitro* pharmacological screens with directly translational outcomes. Altogether, we anticipate that future advances in patient-specific hiPSC-based *in vitro* modeling of NMDs will be instrumental for gaining deeper understanding of human NMD pathophysiology and will lead to streamlined developments of pharmacotherapies for these devastating disorders.

## Author Contributions

ZF, EL, and TC wrote the manuscript. TC and ZF generated figures for the manuscript. AK and NB edited the manuscript. All authors contributed to the article and approved the submitted version.

## Conflict of Interest

The authors declare that the research was conducted in the absence of any commercial or financial relationships that could be construed as a potential conflict of interest.

## Publisher’s Note

All claims expressed in this article are solely those of the authors and do not necessarily represent those of their affiliated organizations, or those of the publisher, the editors and the reviewers. Any product that may be evaluated in this article, or claim that may be made by its manufacturer, is not guaranteed or endorsed by the publisher.
